# Synthesis of *Meso*-Diarylaminocorroles via S_N_Ar Reactions

**DOI:** 10.3390/molecules24030642

**Published:** 2019-02-12

**Authors:** Kento Ueta, Takayuki Tanaka, Atsuhiro Osuka

**Affiliations:** Department of Chemistry, Graduate School of Science, Kyoto University, Kyoto 606-8502, Japan; ueta@kuchem.kyoto-u.ac.jp

**Keywords:** corrole, peripheral substitution, S_N_Ar reaction, UV/Vis absorption, cyclic voltammetry

## Abstract

A corrole is a tetrapyrrolic macrocycle known as a ring-contracted porphyrinoid. Despite the progress of the synthetic chemistry of *meso*-aryl-substituted corroles since the early 2000s, *meso*-heteroatom-substituted corroles have been scarcely reported. Herein we report that the S_N_Ar-type substitution reaction of a *meso*-chlorocorrole silver complex with diphenylamine or carbazole in the presence of NaH as a base produced *meso*-aminocorroles. The structures, ultraviolet–visible spectroscopy (UV/Vis), and emission spectra of these *meso*-aminocorroles were discussed. Furthermore, the oxidation reaction of a *meso*-diphenylaminocorrole was examined, which resulted in the formation of 10,10-diethoxyisocorrole.

## 1. Introduction

**Chart 1:**
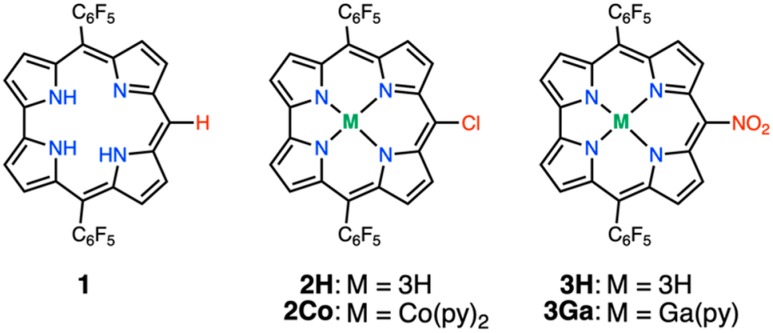
*Meso*-free corrole **1**, *meso*-chlorocorroles **2M** (M = H, Co(py)_2_) and *meso*-nitrocorroles **3M** (M = 3H, Ga(py)).

A corrole is a tetrapyrrolic macrocycle possessing three methine carbons and one direct pyrrole-pyrrole linkage [1,2,3,4,5,6,7,8,9,10,11]. Since the first report in 1965 [12], corroles have been recognized as a unique ring-contracted porphyrinoid because of the inner *3NH*-type structure. The inner cavity of the corrole can serve as a trivalent metal ligand, and many resultant corrole complexes often have the central metals with higher oxidation states, which have been studied as catalysts for various reactions [13,14,15,16,17]. This is a great advantage of corroles against porphyrins that have inner *2NH*-type structure. However, the synthetic chemistry of corroles has been far behind the porphyrin counterpart. The synthesis of *meso*-triaryl-substituted corroles was developed in the early 2000s [18,19,20], while the useful Rothemund–Lindsey type condensation for *meso*-tetraaryl-substituted porphyrins had been invented as early as 1980s [21]. A variety of peripheral functionalizations are now available to create novel porphyrin derivatives mostly thanks to the large scale production of *meso*-free-type porphyrins [22,23]. On the other hand, *meso*-free corroles have been almost unexplored until our recent reports on the effective synthesis of 5,15-bis(pentafluorophenyl)corrole (**1**) (Chart 1) [24,25,26]. The electrophilic substitution reactions on **1** were examined, and *meso*-chlorocorroles **2M** (**2H**: M = 3H, **2Co**: M = Co(py)_2_) and *meso*-nitrocorroles **3M** (**3H**: M = 3H, **3Ga**: M = Ga(py)) were synthesized [27]. As an extension, here we report the first example of *meso*-aminocorroles, which were obtained by nucleophilic aromatic substitution (S_N_Ar) reactions of a *meso*-chlorocorrole silver complex with diphenylamine or carbazole. Since heteroatom-incorporation on the porphyrin scaffolds have been known as an effective way to perturb their electronic properties, the synthetic method described herein will be useful to create novel functional and electronically perturbed corrole derivatives [11].

## 2. Results and Discussion

**Scheme 1:**
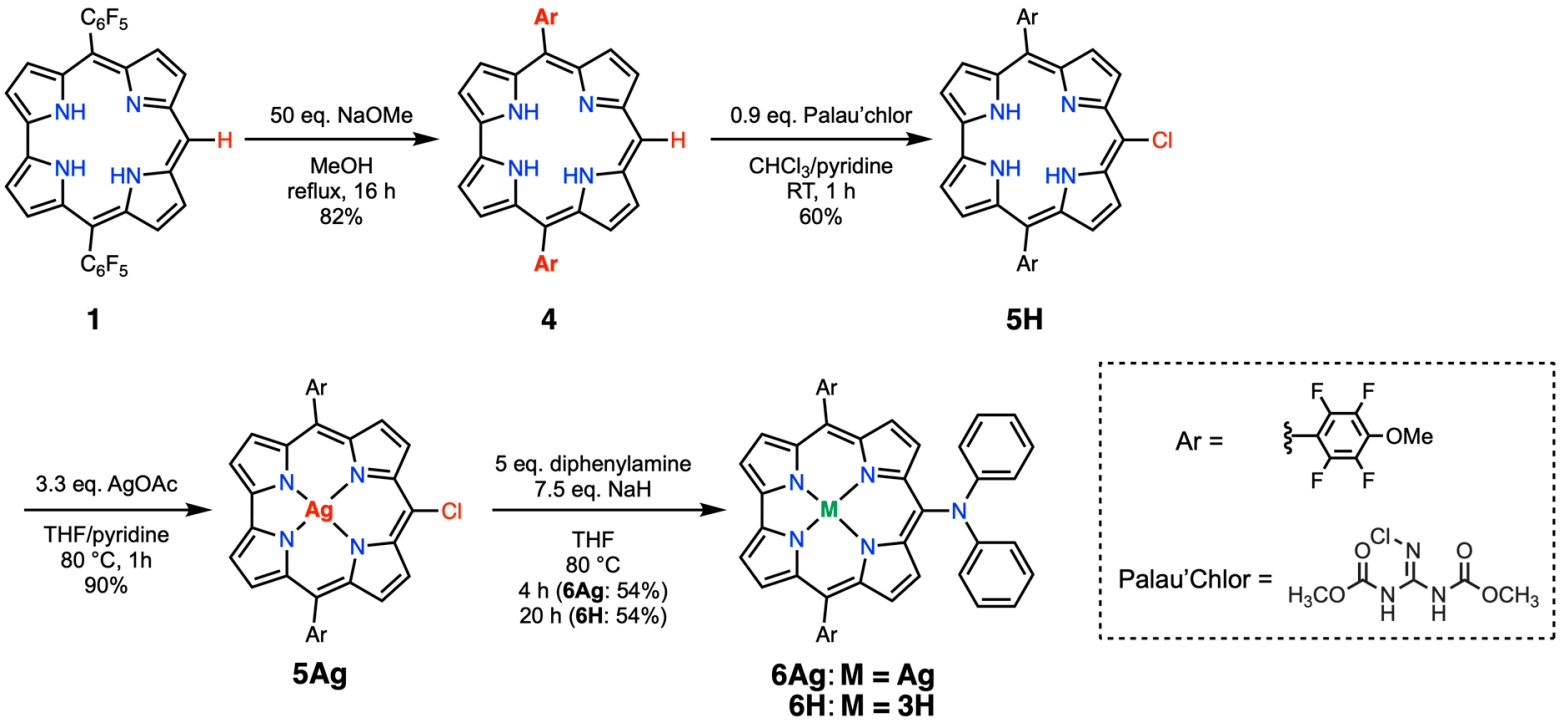
Synthesis of *meso*-diphenylaminocorroles.

Although several methods of S_N_Ar-type reactions against *meso*-haloporphyrins are known in the literature [28,29,30,31,32,33,34], such usual reaction conditions were found to be problematic for corroles having a pentafluorophenyl group as a *meso*-substituent, because the *para*-positions of pentafluorophenyl groups reacted preferentially with nucleophiles [35]. To avoid such an undesirable reaction, the *para*-positions of the pentafluorophenyl groups of **1** were first reacted with a large excess of sodium methoxide in refluxing methanol to give methoxy adduct **4** (Scheme 1). Then, **4** was chlorinated with Palau’Chlor in chloroform/pyridine at room temperature to afford *meso*-chlorocorrole **5H** in 60% yield [26]. 

The S_N_Ar reaction of **5H** was examined under various conditions, but we only isolated a trace amount of an adduct when diphenylamine was used in the presence of sodium hydride as a base. Under these conditions, one of the NH-sites of **5H** was smoothly deprotonated, preventing substitution reaction at the *meso*-position [6,36]. Thus, the silver complex **5Ag** was prepared by the standard metalation reaction [37]. Silver corrole **5Ag** was reacted with five equivalents of diphenylamine in the presence of sodium hydride at 80 °C in tetrahydrofuran (THF) for 4 h, which gave *meso*-diphenylaminocorrole **6Ag** in 54% yield. Under these conditions, silver corroles are known to be demetalated with an excess amount of sodium hydride, thus giving the corresponding freebase **6H** in 54% yield when the same reaction was run for 20 h [37].

**Figure 1:**
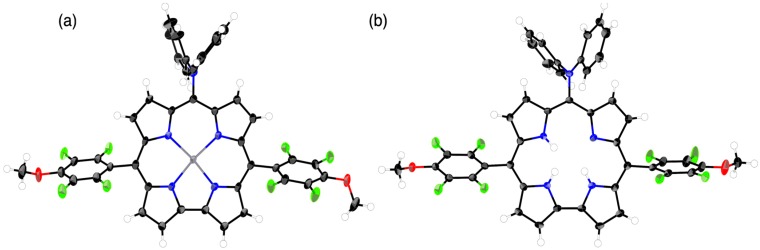
X-Ray crystal structures of (**a**) **6Ag** and (**b**) **6H**. The thermal ellipsoids were scaled to 50% probability. Solvent molecules were omitted for clarity.

The ^1^H-NMR spectra of **6Ag** and **6H** exhibited signals attributable to four β-protons, three phenyl protons, and methoxy proton. The ^19^F-NMR spectra showed two doublets due to the 4-methoxy-2,3,5,6-tetrafluorophenyl groups in the range from −139 to −158 ppm. The structures were unambiguously revealed by X-ray diffraction analysis (Figure 1). The mean-planes defined by the diphenylamino segments were tilted by 63.8–69.2° from the mean-planes of the corrole cores. The C*_meso_*—N bond lengths were 1.431(3) Å for **6Ag** and 1.433(2) Å for **6H**, which were slightly shorter than that of *meso*-nitrocorrole gallium(III) complex **3Ga** (1.46 Å) [27].

**Scheme 2:**
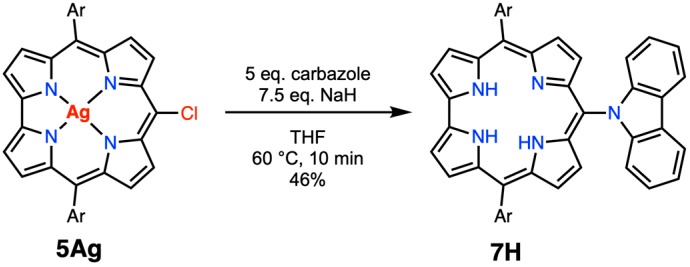
Synthesis of *meso*-carbazolylcorrole.

The same substitution reaction was conducted using carbazole as a nucleophile (Scheme 2). In this case, the reaction conditions were slightly modified. In a round-bottomed flask, five equivalents of carbazole were dissolved in THF and deprotonated by addition of 7.5 equivalents of sodium hydride, which was slowly added to a THF solution of **5Ag** at 80 °C. The reaction mixture was stirred for 10 min. After separation by silica gel column chromatography, *meso*-carbazolylcorrole **7H** was obtained in 46% yield. In this case, only a trace amount of silver complex remained in the reaction mixture. The ^1^H-NMR spectrum of **7H** exhibited a similar spectral pattern to that of **6H** except for four peaks due to the carbazole segment. This new method is potentially useful to produce any *meso*-heteroatom-substituted corroles, although the choice of the base may be crucial to suppress demetalation before reactions.

**Figure 2:**
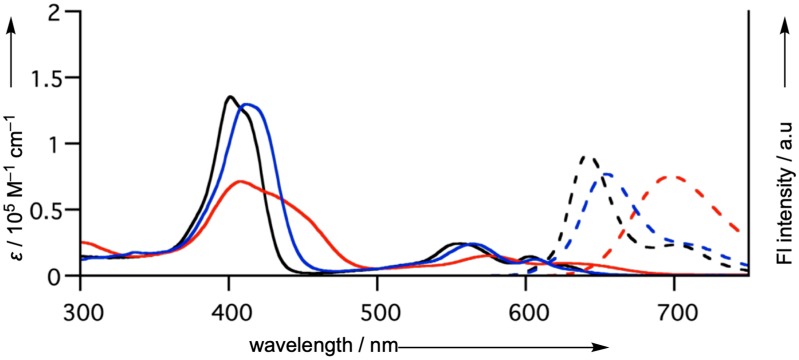
Ultraviolet–visible (UV/Vis) absorption (solid line) and emission (broken line) spectra of **6H** (red), **7H** (blue), and **4** (black) in CH_2_Cl_2_.

Incorporation of heteroatoms at the *meso*-position of porphyrinoids is known to induce electronic perturbation on the optical and electrochemical properties. The ultraviolet–visible (UV/Vis) absorption spectrum of **6H** in dichloromethane indeed showed a broader Soret-band at 408 nm and red-shifted Q-like bands reaching to 700 nm (Figure 2). These spectral characteristics could be understood in terms of well-perturbed molecular orbital profiles. The density functional theory (DFT) calculation for **6H** at the level of B3LYP/6-311G(d,p) revealed the large splitting of the HOMO and HOMO–2, in which the diphenylamino segments have significant orbital coefficients, while the LUMO and LUMO+1 remained mostly intact as compared with those of **4**. Because the a_2_-like HOMO of corroles has a large coefficient at the *meso*-position, the effective perturbation from the diphenylamino group raised the HOMO energy of **6H**, giving rise to the decreased HOMO–LUMO gap. In contrast, *meso*-carbazole-substituted corrole **7H** exhibited absorption features similar to **4**, rather than **6H**. Indeed, the DFT calculation implied negligible effects of the carbazolyl moiety on the HOMO of **7H**, presumably due to the lower HOMO energy level of the carbazole as well as a larger dihedral angle (ca. 82°) between the carbazole unit and the corrole plane (Figure S3-6). A cyclic voltammogram of **6H** in CH_2_Cl_2_ showed reversible oxidation and irreversible reduction waves at 0.07 and −1.58 V, respectively, versus the ferrocene/ferrocenium couple (Fc/Fc^+^), while **7H** showed the first oxidation and reduction waves at 0.48 and −1.42 V as reversible peaks. The large cathodic shift of the first oxidation wave in **6H** was consistent with the raised HOMO, as indicated by the DFT calculation. The electrochemical HOMO-LUMO gap of **6H** (1.65 eV) was relatively smaller than those of **4** (1.73 eV) and **7H** (1.90 eV) in accordance with the observed UV/Vis absorption red-shift. Notably, the absorption and emission spectra of **6H** and **7H** were weakly solvent dependent (Figures S3-5 and S3-6). The absorption spectral changes in polar solvents might have been due to the contribution of protonated/deprotonated corrole species [27]. The emission peaks of **6H** were slightly red-shifted in polar solvents with Stokes shifts of 1050–2236 cm^−1^, while the emission peaks of **7H** were almost unchanged with Stokes shifts of 1066–1354 cm^−1^, which indicated that the structural reorganization in the excited state was more significant in **6H**. Nevertheless, they were unlikely to exhibit twisted intramolecular charge transfer (TICT)-like behaviors as seen in the *meso*-nitrocorroles **3H** and **3Ga**, and *meso*-arylaminosubporphyrins [27,38,39,40]. The optical properties of **4** to **8** are summarized in Table 1.

**Scheme 3:**
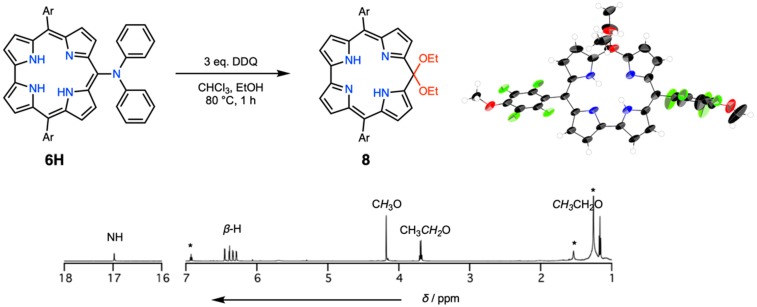
Attempted oxidation of **6H**. The X-ray crystal structure of **8** was shown in the right (thermal ellipsoids: 50%, solvent molecules were omitted for clarity). The ^1^H-NMR spectrum of **8** in CDCl_3_ is shown below.

Heteroatom-embedded π-extended porphyrinoids have shown more perturbed electronic and optical properties as compared with heteroatom-substituted analogs. Thus, the next target may be heteroatom-embedded fused corroles [32,40,41,42,43,44]. Along with this line, diphenylamine-substituted corrole **6H** was subjected to the conditions previously reported to synthesize diphenylamine-fused porphyrins, but all the attempts to obtain fused products failed. However, when the oxidation was examined with 2,3-dichloro-5,6-dicyano-1,4-benzoquinone (DDQ) in chloroform/ethanol at 80 °C for 1 h, a brown fraction was isolated by silica gel column chromatography, which showed its molecular ion peak at *m*/*z*: 742.1811 (calculated for [C_37_H_26_N_4_O_4_F_8_]^+^; M^+^, *m*/*z*: 742.1821) in HR-APCI-TOF-MS (Scheme 3). Fortunately, a single crystal of the compound was obtained by slow vapor diffusion of *n*-hexane into its dichloromethane solution. Unexpectedly, the structure was revealed by X-ray diffraction analysis to be 10,10-diethoxyisocorrole **8** [45,46,47]. In **8**, the isocorrole ligand served as a divalent ligand and the intramolecular hydrogen-bonding interaction was now more favorable. The ^1^H-NMR spectrum showed an NH peak at 16.98 ppm, four doublets of β-protons at 6.45, 6.39, 6.34, and 6.29 ppm, and one triplet and one quartet peaks at 1.17 and 3.69 ppm, respectively, being attributable to the ethoxy groups. Regarding to the reaction mechanism, we thought that an imine intermediate was formed initially under the oxidative conditions. The imine intermediate was attacked by the surrounding nucleophiles such as ethanol. Finally, the elimination of diphenylamine and consecutive addition of another ethanol would yield isocorrole **8** (Figure S7-1).

## 3. Conclusions

In summary, we synthesized *meso*-diarylaminocorroles, as the first example of *meso*-aminated corroles, by nucleophilic aromatic substitution of a *meso*-chlorocorrole silver complex with diphenylamine or carbazole, and the obtained *meso*-arylaminocorroles were characterized by means of optical and electrochemical measurements. The electronic states of *meso*-diphenylaminocorrole **6H** were more strongly perturbed by the diphenylamino substituent as compared with **7H**, because the flexible aryl groups enabled effective conjugation between the corrole and diphenylamine moieties. An oxidative fusion reaction of **6H** was examined with DDQ, which unexpectedly delivered isocorrole **8** in moderate yield. The nucleophilic aromatic substitution to a corrole ring is unprecedented, to our knowledge, which offers a novel approach to *meso*-functionalized corroles as a fundamental tool for corrole chemistry. Further functionalization on the *meso*-position of corroles is ongoing in our laboratory.

## 4. Materials and Methods 

Commercially available solvents and reagents were used without further purification unless otherwise noted. The spectroscopic grade solvents were used for all the spectroscopic studies. Silica gel column chromatography was performed on Wakogel C-300 (FUJIFILM Wako Pure Chemical Corportaion, 1-2, Doshomachi 3-chome, Chuo-ku, Osaka, Japan). The UV/Vis absorption spectra were recorded on a Shimadzu UV-3600 spectrometer (Shimadzu Corporation, 1, Nishinokyo Kuwabara-cho, Nakagyo-ku, Kyoto, Japan). The fluorescence spectra were recorded on a JASCO spectrofluorometer FP-8500 (JASCO Corporation, 2967-5, Ishikawa-cho, Hachioji-shi, Tokyo, Japan). The ^1^H- and ^19^F-NMR spectra were recorded on a JEOL ECA-600 spectrometer (JEOL Ltd., 3-1-2 Musashino, Akishima-shi, Tokyo, JAPAN) (operating as 600.17 MHz for ^1^H and 564.73 MHz for ^19^F) using the residual solvent as an internal reference for ^1^H (δ = 7.26 ppm in CDCl_3_) and hexafluorobenzene as an external reference for ^19^F (δ = −162.9 ppm). HR-APCI-TOF-MS was conducted on a BRUKER micrOTOF model using positive ion mode. The redox potentials were measured by cyclic voltammetry on an ALS electrochemical analyzer model 612E. The single-crystal X-ray diffraction analysis data were collected at −180 °C with a Rigaku XtaLAB P200 (Rigaku Corporation, 3-9-12, Matsubara-cho, Akishima-shi, Tokyo, Japan) by using graphite monochromated Cu-*Kα* radiation (*λ* = 1.54187 Å). The structures were solved by direct methods (SHELXT-2014/5) [48] and refined with the full-matrix least-squares technique (SHELXL-2014/7) [49,50]. All calculations were carried out using the Gaussian 16 program

### 4.1. Synthesis of ***4***

The *meso*-free corrole **1** (0.30 mmol, 184 mg) and sodium methoxide (15 mmol, 810 mg, 50 equivalent) were dissolved in MeOH (30 mL, 10 mM). The reaction mixture was stirred at 80 °C for 16 h, and then neutralized with aqueous HCl. The crude product was extracted with dichloromethane. The organic layer was washed with brine and dried over anhydrous Na_2_SO_4_. After the solvent was removed under reduced pressure, the residue was purified with a short silica gel column using dichloromethane as an eluent. Recrystallization from dichloromethane/*n*-hexane gave corrole **4** (161 mg, 0.25 mmol, 82%).

^1^H-NMR (600 MHz, CDCl_3_, 25 °C) δ / ppm = 9.69 (s, 1H, *meso*-H), 9.09 (br, 4H, β-H), 8.84 (d, *J* = 4.1 Hz, 2H, β-H), 8.60 (brs, 2H, β-H), and 4.39 (s, 6H, OMe). ^19^F-NMR (585 MHz, CDCl_3_, 25°C) δ / ppm = −139.77 (s, 4F, *o*-F), and −157.86 (s, 4F, *m*-F). HR-APCI-TOF-MS *m*/*z* = 654.1274 (calculated for [C_33_H_18_N_4_O_2_F_8_]^+^; M^+^, *m*/*z* = 654.1297).

### 4.2. Synthesis of ***5H***

Palau’Chlor (90 μmol, 19 mg, 0.90 equiv.) was added to a solution of corrole **4** (0.10 mmol, 69 mg) in chloroform/pyridine (35 mL/0.35 mL, 3 mM). The reaction mixture was stirred for 1 h at room temperature and passed through a short silica pad. After the solvent was removed under reduced pressure, the residue was purified by silica gel column chromatography using *n*-hexane/dichloromethane (*v*/*v* = 7:3) as an eluent. Recrystallization from dichloromethane/*n*-hexane gave *meso*-chlorocorrole **5H** (41 mg, 60 μmol, 60%).

^1^H-NMR (600 MHz, CDCl_3_, 25 °C) δ / ppm = 9.40 (d, *J* = 4.6 Hz, 2H, β-H), 9.08 (d, *J* = 3.7 Hz, 2H, β-H), 8.81 (d, *J* = 4.6 Hz, 2H, β-H), 8.57 (brs, 2H, β-H), and 4.40 (s, 6H, OMe). ^19^F-NMR (585 MHz, CDCl_3_, 25 °C) δ / ppm = −139.75 (s, 4F, *o*-F), and −157.69 (s, 4F, *m*-F). HR-APCI-TOF-MS *m*/*z* = 689.0962 (calculated for [C_33_H_18_N_4_O_2_^35^ClF_8_]^+^; [M + H]^+^, *m*/*z* = 689.0985).

### 4.3. Synthesis of ***5Ag***

The corrole **5H** (40 μmol, 28 mg) and silver(I) acetate (0.132 mmol, 22 mg, 3.3 equivalent) were dissolved in THF/pyridine (4 mL/2 mL). The reaction mixture was gradually heated up to 80 °C, stirred for 1 h, and passed through a Florisil^®^ pad (SIGMA-ALDRICH JAPAN K.K., 2-2-24, Higashishinagawa, Shinagawa-ku, Tokyo, Japan). After the solvent was removed under reduced pressure, the recrystallization from dichloromethane/*n*-hexane gave the corrole Ag(III) complex **5Ag** (28 mg, 36 μmol, 90%).

^1^H-NMR (600 MHz, CDCl_3_, 25 °C) δ / ppm = 9.53 (d, *J* = 5.0 Hz, 2H, β-H), 9.30 (d, *J* = 4.1 Hz, 2H, β-H), 9.00 (d, *J* = 4.6 Hz, 2H, β-H), 8.77 (d, *J* = 4.1 Hz, 2H, β-H), and 4.42 (s, 6H, OMe). ^19^F-NMR (585 MHz, CDCl_3_, 25 °C) δ / ppm = −139.10 (d, *J* = 17.5 Hz, 4F, *o*-F), and −157.69 (d, *J* = 17.5 Hz, 4F, *m*-F). HR-APCI-TOF-MS *m*/*z* = 791.9715 (calculated for [C_33_H_14_N_4_O_2_^107^Ag^35^ClF_8_]^+^; M^+^, *m*/*z* = 791.9723).

### 4.4. Nucleophilic Aromatic Substitution Reaction of 5Ag with Diphenylamine

Corrole **5Ag** (20 μmol, 15 mg), NaH (0.15 mmol, 6.0 mg, 7.5 equivalent), and diphenylamine (0.10 mmol, 17 mg, 5 equivalent) were placed in a round-bottomed flask, and were dissolved in THF (1 mL, 20 mM). The mixture was stirred for 4 h at 80 °C, and passed through a short silica pad. After the solvent was removed under reduced pressure, the residue was purified by silica gel column chromatography using *n*-hexane/dichloromethane (*v*/*v* = 7:3) as an eluent. The recrystallization from dichloromethane/*n*-hexane gave *meso*-diphenylaminocorrole Ag(III) complex **6Ag** (10 mg, 11 mmol, 54%). When the reaction mixture was stirred for 20 h, demetalated freebase corrole **6H** was obtained (9.0 mg, 11 mmol, 54% yield).

**6Ag**: ^1^H-NMR (600 MHz, CDCl_3_, 25 °C) δ / ppm = 9.24 (d, *J* = 4.6 Hz, 2H, β-H), 9.12 (d, *J* = 4.6 Hz, 2H, β-H), 8.80 (d, *J* = 4.6 Hz, 2H, β-H), 8.71 (d, *J* = 4.6 Hz, 2H, β-H), 7.30 (d, *J* = 7.8 Hz, 4H, *o*-Ph), 7.19 (dd, *J*_1_ = 7.8 Hz, *J*_2_ = 7.3 Hz, 4H, *m*-Ph), 6.92 (t, *J* = 7.3 Hz, 2H, *p*-Ph), and 4.39 (s, 6H, OMe). ^19^F-NMR (565 MHz, CDCl_3_, 25 °C) δ / ppm = −139.05 (d, *J* = 17.5 Hz, 4F, *o*-F), and −157.87 (d, *J* = 17.5 Hz, 4F, *m*-F). HR-APCI-TOF-MS *m*/*z* = 925.0827 (calculated for [C_45_H_24_N_5_O_2_^107^AgF_8_]^+^; M^+^, *m*/*z* = 925.0848).

**6H**: ^1^H-NMR (600 MHz, CDCl_3_, 25 °C) δ / ppm = 9.02 (d, *J* = 4.2 Hz, 4H, β-H), 9.01 (d, *J* = 4.2 Hz, 4H, β-H), 8.64 (d, *J* = 4.2 Hz, 2H, β-H), 8.53 (brs, 2H, β-H), 7.32 (d, *J* = 7.8 Hz, 4H, *o*-Ph), 7.18 (dd, *J*_1_ = 7.8 Hz, *J*_2_ = 7.3 Hz, 4H, *m*-Ph), 6.90 (t, *J* = 7.3 Hz, 2H, *p*-Ph), and 4.37 (s, 6H, MeO). ^19^F-NMR (565 MHz, CDCl_3_, 25 °C) δ / ppm = −139.67 (s, 4F, *o*-F), and −157.84 (s, 4F, *m*-F). HR-APCI-TOF-MS *m*/*z* = 822.2042 (calculated for [C_45_H_28_N_5_O_2_F_8_]^+^; [M + H]^+^, *m*/*z* = 821.2110).

### 4.5. Nucleophilic Aromatic Substitution Reaction of ***5Ag*** with Carbazole

The corrole **5Ag** (20 μmol, 15 mg) was dissolved in THF (0.5 mL), to which a mixture of NaH (0.2 mmol, 8 mg, 10 equivalent) and carbazole (0.1 mmol, 17 mg, 5 equivalent) was added slowly. The mixture was stirred for 10 min at 80 °C, and passed through a short silica pad. After the solvent was removed under reduced pressure, the residue was purified by silica gel column chromatography using *n*-hexane/dichloromethane (*v*/*v* = 7:3) as an eluent. Recrystallization from dichloromethane/*n*-hexane gave *meso*-carbazolylcorrole **7H** (7.5 mg, 9.1 μmol, 46%).

**7H**: ^1^H-NMR (600 MHz, CDCl_3_, 25 °C) δ / ppm = 9.09 (brs, 2H, β-H), 8.60 (brs, 4H, 2×β-H), 8.43 (d, *J* = 7.8 Hz, 2H, Cz), 8.26 (brs, 2H, β-H), 7.40 (t, *J* = 7.8 Hz, 2H, Cz), 7.28 (t, *J* = 7.8 Hz, 2H, Cz), 6.85 (d, *J* = 7.8 Hz, 2H, Cz), and 4.37 (s, 6H, MeO). ^19^F-NMR (565 MHz, CDCl_3_, 25 °C) δ / ppm = −139.75 (s, 4F, *o*-F), and −157.69 (s, 4F, *m*-F). HR-APCI-TOF-MS *m*/*z* = 820.1908 (calculated for [C_45_H_25_N_5_O_2_F_8_]^+^; [M + H]^+^, *m*/*z* = 820.1953).

### 4.6. Synthesis of ***8***

Corrole **6H** (5 μmol, 4.1 mg) was dissolved in CHCl_3_/EtOH (200 mL/1 mL), to which a solution of DDQ (15 mmol, 3.4 mg, 3 equiv.) in CHCl_3_ (4 mL) was added slowly. The mixture was stirred for 1 h at 80 °C, and passed through a short silica pad. After the solvent was removed under reduced pressure, the residue was purified by silica gel column chromatography using *n*-hexane/dichloromethane (*v*/*v* = 7:3) as an eluent. The recrystallization from dichloromethane/*n*-hexane gave 10,10’-diethoxyisocorrole **8** (1.7 mg, 2.3 μmol, 48%).

^1^H-NMR (600 MHz, CDCl_3_, 25 °C) δ / ppm = 16.98 (s, 2H, NH), 6.45 (d, *J* = 4.6 Hz, 2H, β-H), 6.39 (d, *J* = 4.6 Hz, 2H, β-H), 6.34 (d, *J* = 4.6 Hz, 2H, β-H), 6.29 (d, *J* = 4.6 Hz, 2H, β-H), 4.18 (s, 6H, OMe), 3.69 (q, *J* = 6.9 Hz, 4H, OCH_2_CH_3_), and 1.17 (t, *J* = 6.9 Hz, 6H, OCH_2_CH_3_). ^19^F-NMR (565 MHz, CDCl_3_, 25 °C) δ = −140.70 (d, *J* = 17.5 Hz, 4F, *o*-F), and −158.21 (d, *J* = 17.5 Hz, 4F, *m*-F). HR-APCI-TOF-MS *m*/*z* = 742.1811 (calculated for [C_37_H_26_N_4_O_4_F_8_]^+^; M^+^, *m*/*z* = 742.1821).

## Figures and Tables

**Table 1:** Summary of the absorption and emission spectra of **4**, **5H**, **5Ag**, **6H**, **7H**, and **8** in CH_2_Cl_2_.

Compound	Absorption peaks/nm (ε/10^5^ M^−1^cm^−1^)	Fluorescence peaks/nm	Φ_F_, Fluorescence Quantum Yield
**4**	401 (1.35), 556 (0.24), 604 (0.14)	642, 700	9.2%
**5H**	412 (1.71), 566 (0.28), 616 (0.17)	648, 704	2.3%
**5Ag**	423 (1.57), 563 (0.25), 579 (0.34)	-	-
**6H**	408 (0.71), 574 (0.15), 624 (0.09)	699	10.4%
**7H**	411 (1.30), 565 (0.24), 606 (0.12)	654	9.2%
**8**	418 (0.28), 720 (0.02)	-	-

Note: Fluorescence spectra were recorded for the dilute solutions by excitation at 400 nm.

## Supplementary Materials

The following are available online. Figure S1: ^1^H and ^19^F-NMR spectra, Figure S2: Observed (top) and simulated (bottom) HR-APCI-TOF-MS, Figure S3: UV/Vis absorption and fluorescence spectra, Figure S4: X-Ray structures, Figure S5: Cyclic voltammogram, Figure S6: Molecular orbital (MO) energy diagrams and Kohn–Sham orbital representations calculated at the B3LYP/6-311G(d,p) level, Scheme S7-1: Plausible reaction mechanism for the formation of **8**. Table S1: Absorption and emission details of **4**, **5**, **6H**, and **7H** in various solvents, Table S2: The photophysical parameters of **6H** and **7H** in various solvents. Table S3: Crystallographic details of **6Ag**, **6H**, and **8**.

## Abbreviations

NMR	Nuclear magnetic resonance
DFT	Density functional theory
HOMO	Highest occupied molecular orbital
LUMO	Lowest occupied molecular orbital
DDQ	2,3-dichloro-5,6-dicyano-1,4-benzoquinone
THF	Tetrahydrofuran
TICT	Twisted intramolecular charge transfer
HR-APCI-TOF-MS	High-resolution atmospheric-pressure-chemical-ionization time-of-flight mass-spectrometry
MO	Molecular orbital
