# Supplementary material for: Synthesis of Meso-Diarylaminocorroles via SNAr Reactions
Source: Molecules. 2019 Feb 12;24(3):642. doi: 10.3390/molecules24030642 (PMC6384549; doi:10.3390/molecules24030642)
Supplement: Supplementary file 1 [file molecules-24-00642-s001.pdf]

Supporting Information

Contents

1. **NMR Spectra**

2. **Mass Spectra**

3. **UV/Vis Absorption and Fluorescence Spectra**

4. **X-Ray Crystallographic Details**

5. **Cyclic Voltammograms**

6. **DFT Calculations**

7. **Plausible Reaction Mechanism for the Formation of 8**

8. **References**

General information

Commercially available solvents and reagents were used without further purification unless otherwise noted. The spectroscopic grade solvents were used for all the spectroscopic studies. Silica gel column chromatography was performed on Wakogel C-300. The UV/Vis absorption spectra were recorded on a Shimadzu UV-3600 spectrometer. The fluorescence spectra were recorded on a JASCO spectrofluorometer FP-8500. The absolute fluorescence quantum yields were determined on a HAMAMATSU C9920–02S. The fluorescence lifetime was recorded on Hamamatsu Photonics Quantaurus-Tau C11367. The ^{1}H and ^{19}F NMR spectra were recorded on a JEOL ECA-600 spectrometer (operating as 600.17 MHz for ^{1}H and 564.73 MHz for ^{19}F) using the residual solvent as an internal reference for ^{1}H (δ = 7.26 ppm in $CDCl_3$) and hexafluorobenzene as an external reference for ^{19}F (δ = –162.9 ppm). High-resolution atmospheric-pressure-chemical-ionization time-of-flight mass-spectrometry (HR-APCI-TOF-MS) was recorded on a BRUKER micrOTOF model using positive ion mode. The redox potentials were measured by cyclic voltammetry on an ALS electrochemical analyzer model 612E. The Single-crystal X-ray diffraction analysis data were collected at –180 °C with a Rigaku XtaLAB P200 by using graphite monochromated Cu-$K\alpha$ radiation (λ = 1.54187 Å). The structures were solved by direct methods (SHELXT-2014/5)[S1,S2] and refined with the full-matrix least-squares technique (SHELXL-2014/7) [S3] . All calculations were carried out using the Gaussian 16 program [S4].

1. NMR Spectra

5,15-Bis(4-methoxy-2,3,5,6-tetrafluorophenyl)corrole **4**

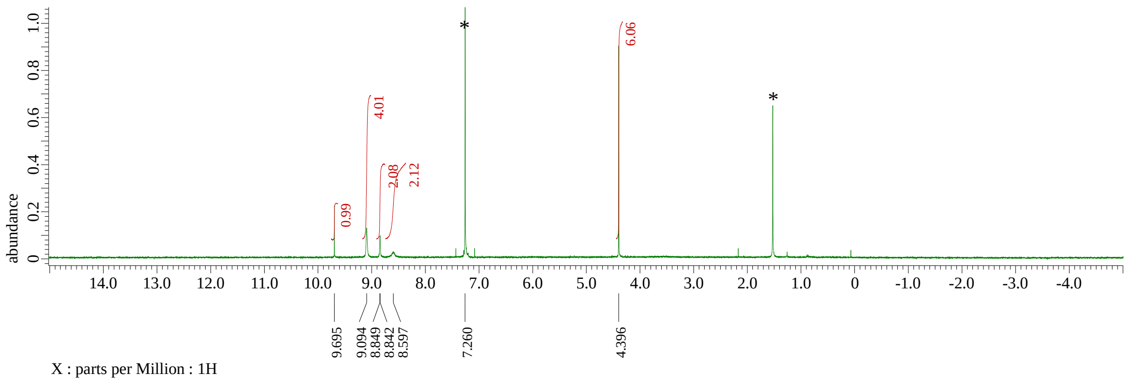

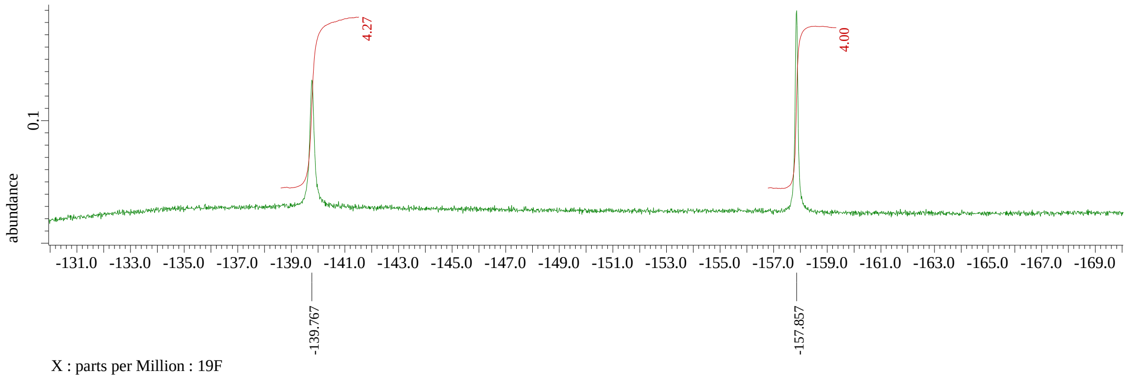

Figure S1-1. ^{1}H and ^{19}F NMR spectra of **4** at 25 °C in $CDCl_3$. *Solvent and impurities.

^{1}H NMR (600 MHz, $CDCl_3$, 25°C) δ / ppm = 9.69 (s, 1H, *meso*-H), 9.09 (br, 4H, β-H), 8.84 (d, *J* = 4.1 Hz, 2H, β-H), 8.60 (brs, 2H, β-H), and 4.39 (s, 6H, OMe).

^{19}F NMR (585 MHz, $CDCl_3$, 25°C) δ / ppm = –139.77 (s, 4F, *o*-F), and –157.86 (s, 4F, *m*-F).

10-Chloro-5,15-bis(4-methoxy-2,3,5,6-tetrafluorophenyl)corrole **5H**

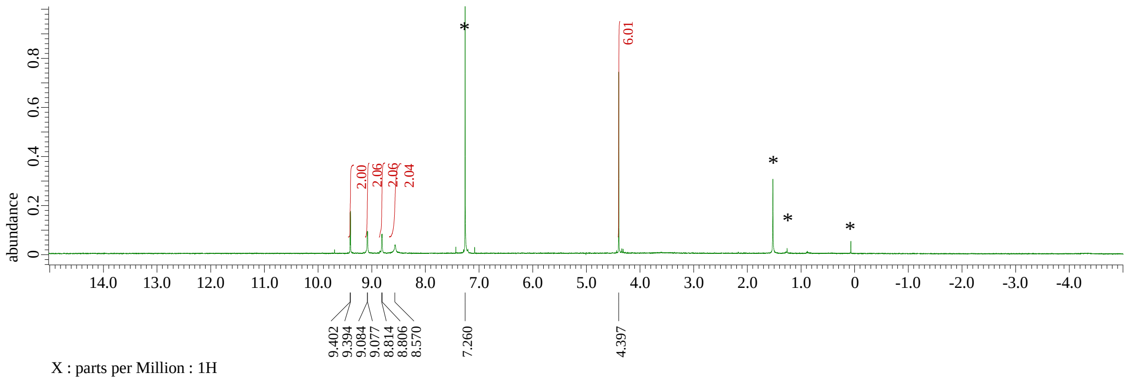

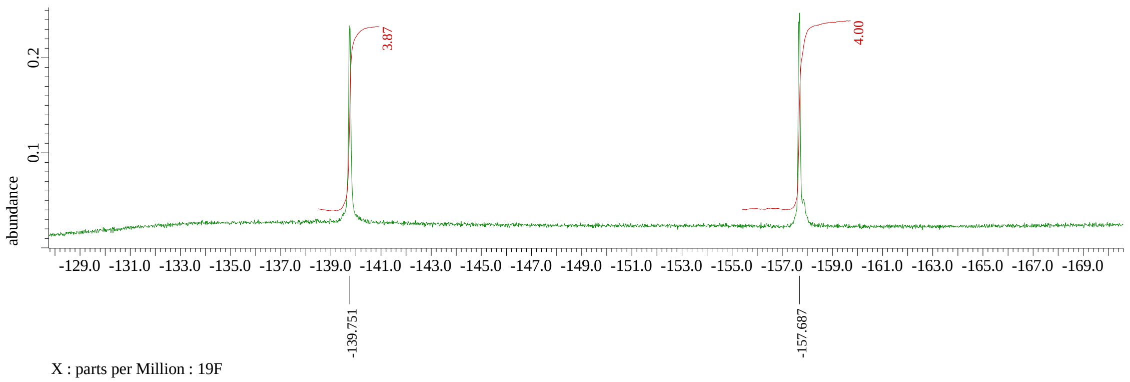

Figure S1-2. ^{1}H and ^{19}F NMR spectra of **5H** at 25 °C in $CDCl_3$. *Solvent and impurities.

^{1}H NMR (600 MHz, $CDCl_3$, 25°C) δ / ppm = 9.40 (d, *J* = 4.6 Hz, 2H, β-H), 9.08 (d, *J* = 3.7 Hz, 2H, β-H), 8.81 (d, *J* = 4.6 Hz, 2H, β-H), 8.57 (brs, 2H, β-H), and 4.40 (s, 6H, OMe).

^{19}F NMR (585 MHz, $CDCl_3$, 25°C) δ / ppm = –139.75 (s, 4F, *o*-F), and –157.69 (s, 4F, *m*-F).

10-Chloro-5,15-bis(4-methoxy-2,3,5,6-tetrafluorophenyl)corrolato silver(III) **5Ag**

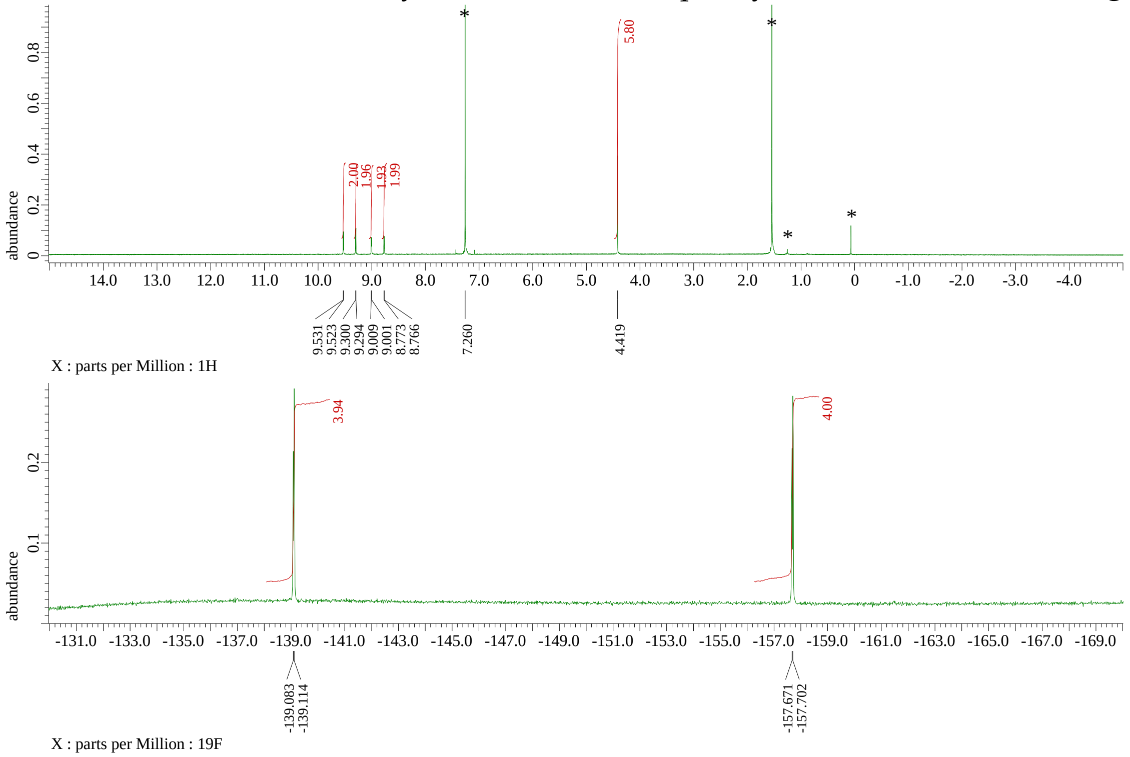

Figure S1-3. ¹H and ¹⁹F NMR spectra of **5Ag** at 25 °C in $CDCl_3$. *Solvent and impurities.

¹H NMR (600 MHz, $CDCl_3$, 25 °C) δ / ppm = 9.53 (d, *J* = 5.0 Hz, 2H, β-H), 9.30 (d, *J* = 4.1 Hz, 2H, β-H), 9.00 (d, *J* = 4.6 Hz, 2H, β-H), 8.77 (d, *J* = 4.1 Hz, 2H, β-H), and 4.42 (s, 6H, OMe).

¹⁹F NMR (585 MHz, $CDCl_3$, 25°C) δ / ppm = –139.10 (d, *J* = 17.5 Hz, 4F, *o*-F), and –157.69 (d, *J* = 17.5 Hz, 4F, *m*-F).

10-Diphenylamino−5,15-bis(4-methoxy-2,3,5,6-tetrafluorophenyl)corrolato silver(III) **6Ag**

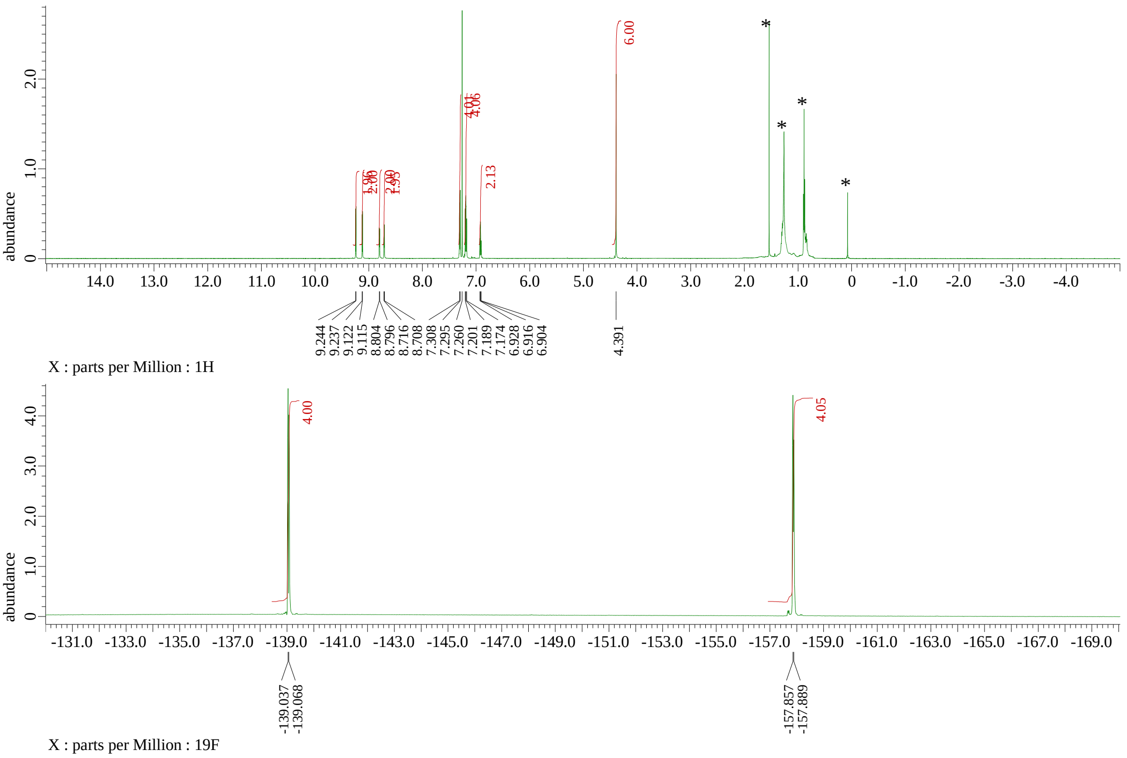

Figure S1-4. ^{1}H and ^{19}F NMR spectra of **6Ag** at 25 °C in $CDCl_3$. *Solvent and impurities.

^{1}H NMR (600 MHz, $CDCl_3$, 25 °C) δ / ppm = 9.24 (d, *J* = 4.6 Hz, 2H, β-H), 9.12 (d, *J* = 4.6 Hz, 2H, β-H), 8.80 (d, *J* = 4.6 Hz, 2H, β-H), 8.71 (d, *J* = 4.1 Hz, 2H, β-H), 7.30 (d, *J* = 7.8 Hz, 4H, *o*-Ph), 7.19 (t, *J* = 8.0 Hz, 4H, *m*-Ph), 6.92 (t, *J* = 7.3 Hz, 2H, *p*-Ph), and 4.39 (s, 6H, OMe).

^{19}F NMR (565 MHz, $CDCl_3$, 25 °C) δ / ppm = –139.05 (d, *J* = 17.5 Hz, 4F, *o*-F), and –157.87 (d, *J* = 17.5 Hz, 4F, *m*-F).

10-Diphenylamino-5,15-bis(4-methoxy-2,3,5,6-tetrafluorophenyl)corrole **6H**

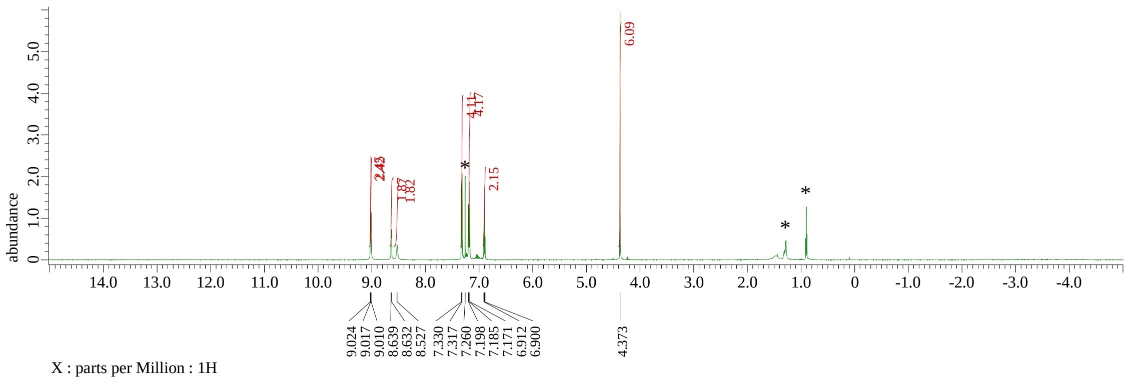

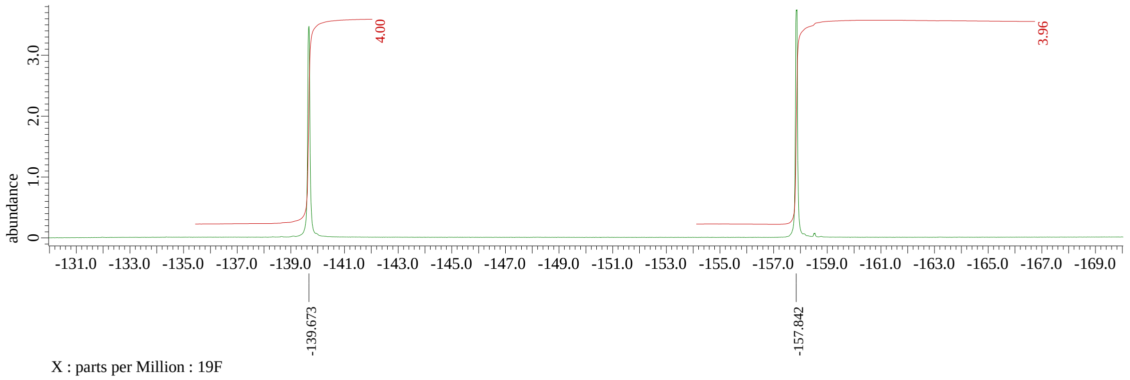

Figure S1-5. ^{1}H and ^{19}F NMR spectra of **6H** at 25 °C in $CDCl_3$. *Solvent and impurities.

^{1}H NMR (600 MHz, $CDCl_3$, 25 °C) δ / ppm = 9.02 (d, *J* = 4.2 Hz, 4H, β-H), 9.01 (d, *J* = 4.2 Hz, 4H, β-H), 8.64 (d, *J* = 4.2 Hz, 2H, β-H), 8.53 (brs, 2H, β-H), 7.32 (d, *J* = 7.8 Hz, 4H, *o*-Ph), 7.18 (t, *J* = 7.8 Hz, 4H, *m*-Ph), 6.90 (t, *J* = 7.3 Hz, 2H, *p*-Ph), and 4.37 (s, 6H, MeO).

^{19}F NMR (565 MHz, $CDCl_3$, 25 °C) δ / ppm = –139.67 (s, 4F, *o*-F), and –157.84 (s, 4F, *m*-F).

10-Carbazolyl-5,15-bis(4-methoxy-2,3,5,6-tetrafluorophenyl)corrole **7H**

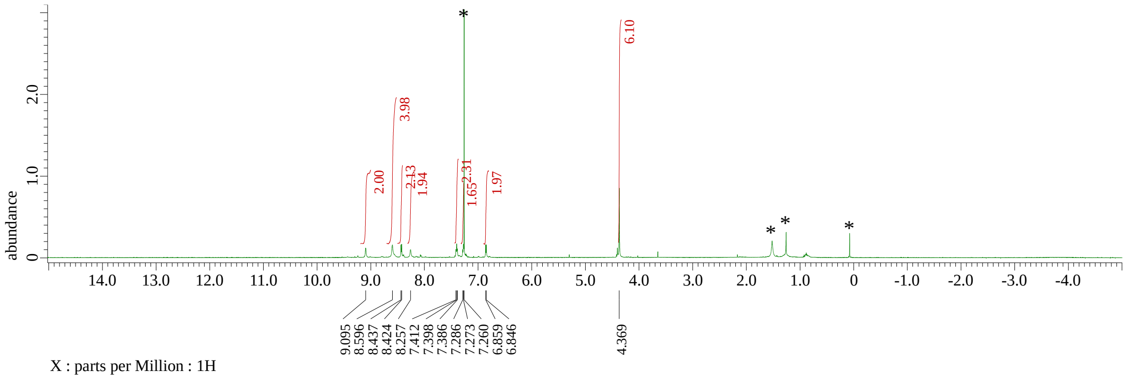

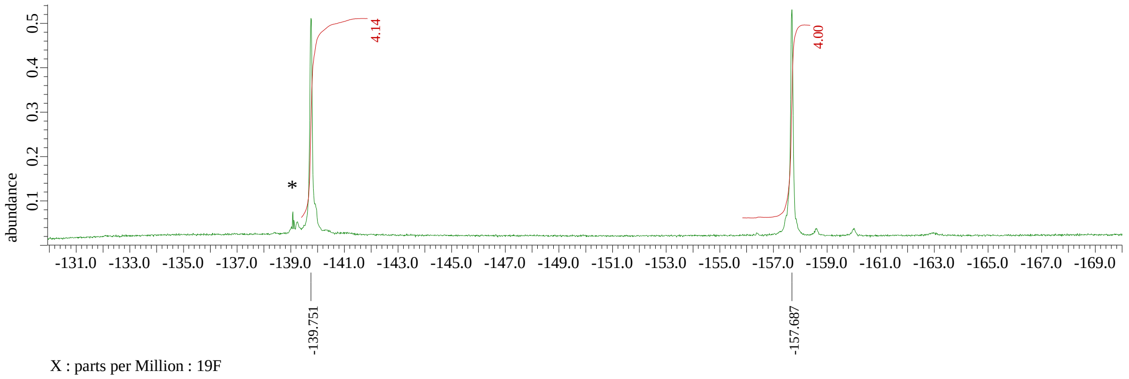

Figure S1-6. ^{1}H and ^{19}F NMR spectra of **7H** at 25 °C in $CDCl_3$. *Solvent and impurities.

^{1}H NMR (600 MHz, $CDCl_3$, 25 °C) δ / ppm = 9.09 (brs, 2H, β-H), 8.60 (brs, 4H, β-H+Cz), 8.43 (d, J = 7.8 Hz, 2H, β-H), 8.26 (brs, 2H, β-H), 7.40 (t, J = 7.6 Hz, 2H, Cz), 7.28 (d, J = 7.3 Hz, 2H, Cz), 6.85 (d, J = 8.3 Hz, 2H, Cz), and 4.37 (s, 6H, MeO).
^{19}F NMR (565 MHz, $CDCl_3$, 25 °C) δ / ppm = –139.75 (s, 4F, *o*-F), and –157.69 (s, 4F, *m*-F).

10,10-Diethoxy-5,15-bis(4-methoxy-2,3,5,6-tetrafluorophenyl)isocorrole **8**

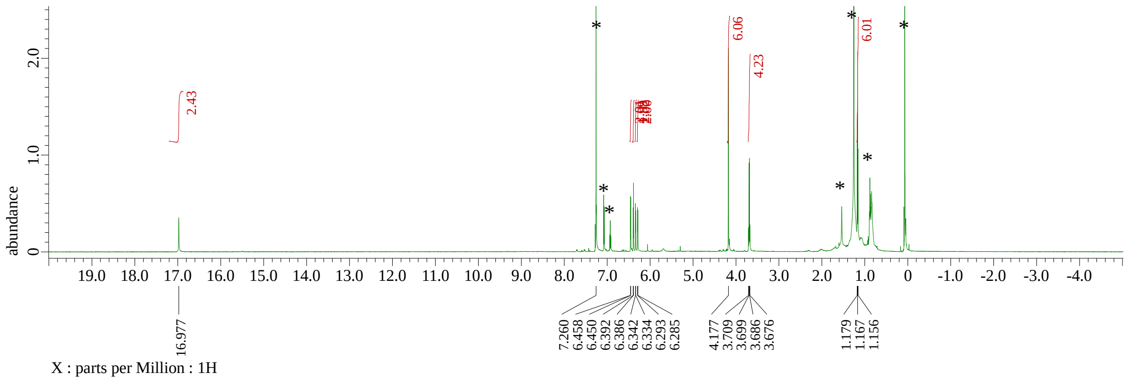

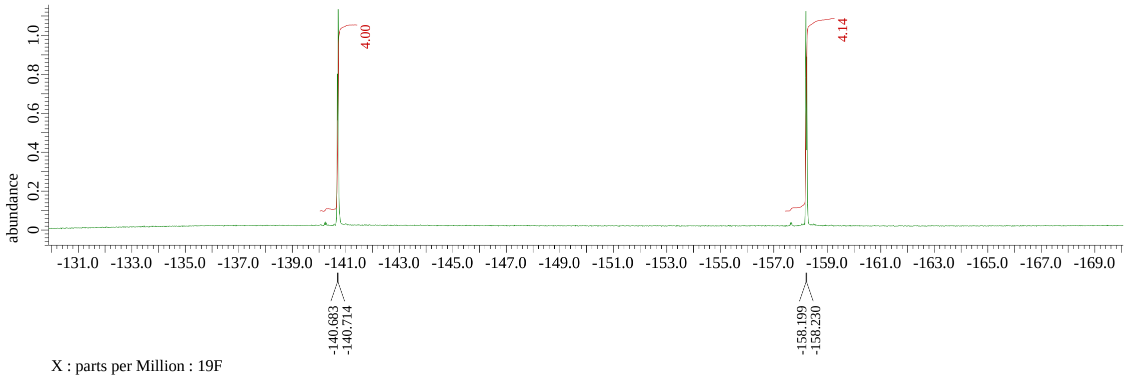

Figure S1-7. ^{1}H and ^{19}F NMR spectra of **8** at 25 °C in $CDCl_3$. *Solvent and impurities.

^{1}H NMR (600 MHz, $CDCl_3$, 25 °C) δ / ppm = 16.98 (s, 2H, NH), 6.45 (d, *J* = 4.6 Hz, 2H, β-H), 6.39 (d, *J* = 4.6 Hz, 2H, β-H), 6.34 (d, *J* = 4.6 Hz, 2H, β-H), 6.29 (d, *J* = 4.6 Hz, 2H, β-H), 4.18 (s, 6H, OMe), 3.69 (q, *J* = 6.9 Hz, 4H, O<u>CH$_2$</u>CH$_3$), and 1.17 (t, *J* = 6.9 Hz, 6H, OCH$_2$<u>CH$_3$</u>).

^{19}F NMR (565 MHz, $CDCl_3$, 25 °C) δ = –140.70 (d, *J* = 17.5 Hz, 4F, *o*-F), and –158.21 (d, *J* = 17.5 Hz, 4F, *m*-F).

2. Mass Spectra

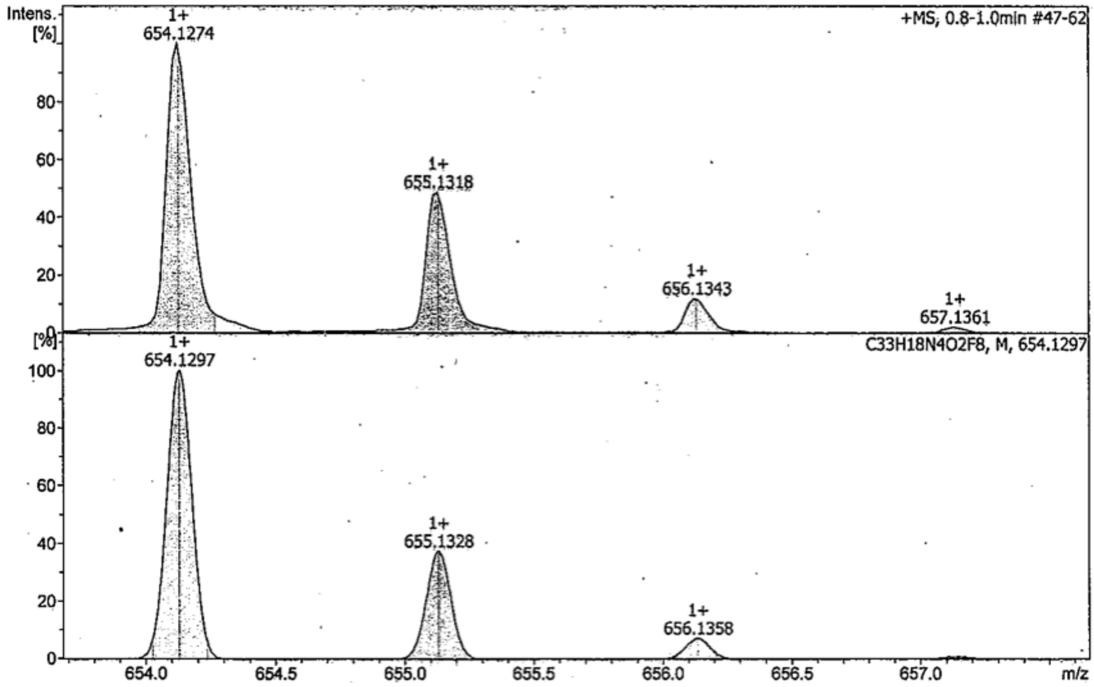

Figure S2-1. Observed (top) and simulated (bottom) HR-APCI-TOF-MS of **4** m/z = 654.1274 (calculated for $[C_{33}H_{18}N_4O_2F_8]^+$; $[M]^+$, m/z = 654.1297).

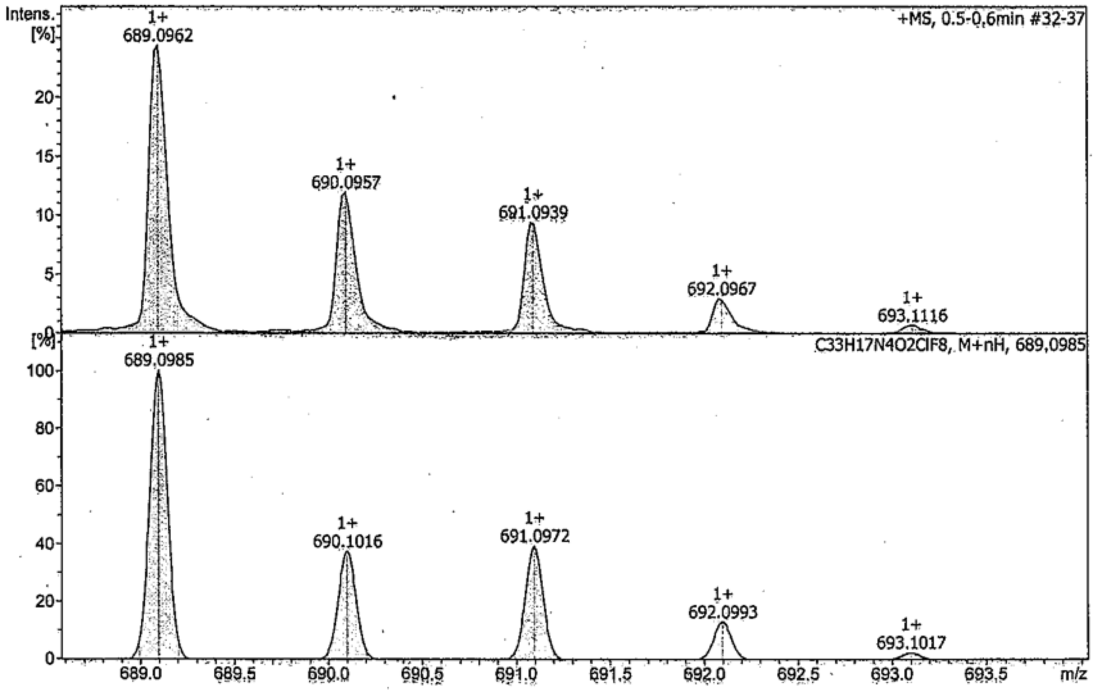

Figure S2-2. Observed (top) and simulated (bottom) HR-APCI-TOF-MS of **5H** m/z = 689.0962 (calculated for $[C_{33}H_{18}N_4O_2{}^{35}ClF_8]^+$; $[M+H]^+$, m/z = 689.0985).

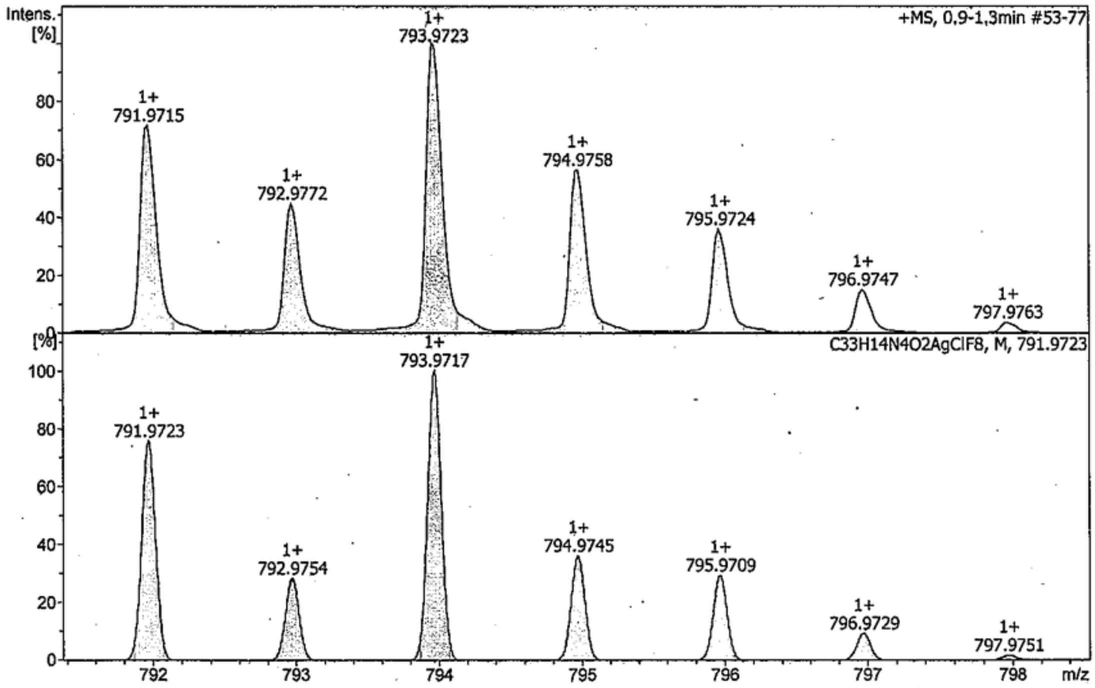

Figure S2-3. Observed (top) and simulated (bottom) HR-APCI-TOF-MS of **5Ag** m/z = 791.9715 (calculated for [$C_{33}H_{14}N_4O_2{}^{107}Ag^{35}ClF_8$]$^+$; [$M$]$^+$, m/z = 791.9723).

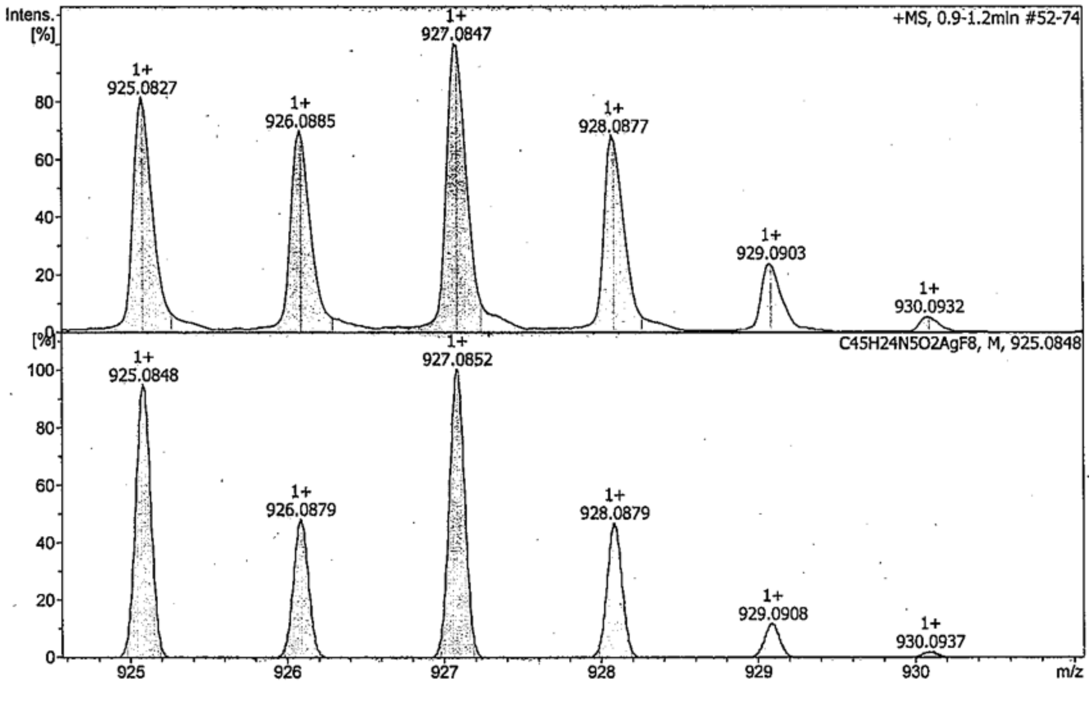

Figure S2-4. Observed (top) and simulated (bottom) HR-APCI-TOF-MS of **6Ag** m/z = 925.0827 (calculated for [$C_{45}H_{24}N_5O_2{}^{107}AgF_8$]$^+$; [$M$]$^+$, m/z = 925.0848).

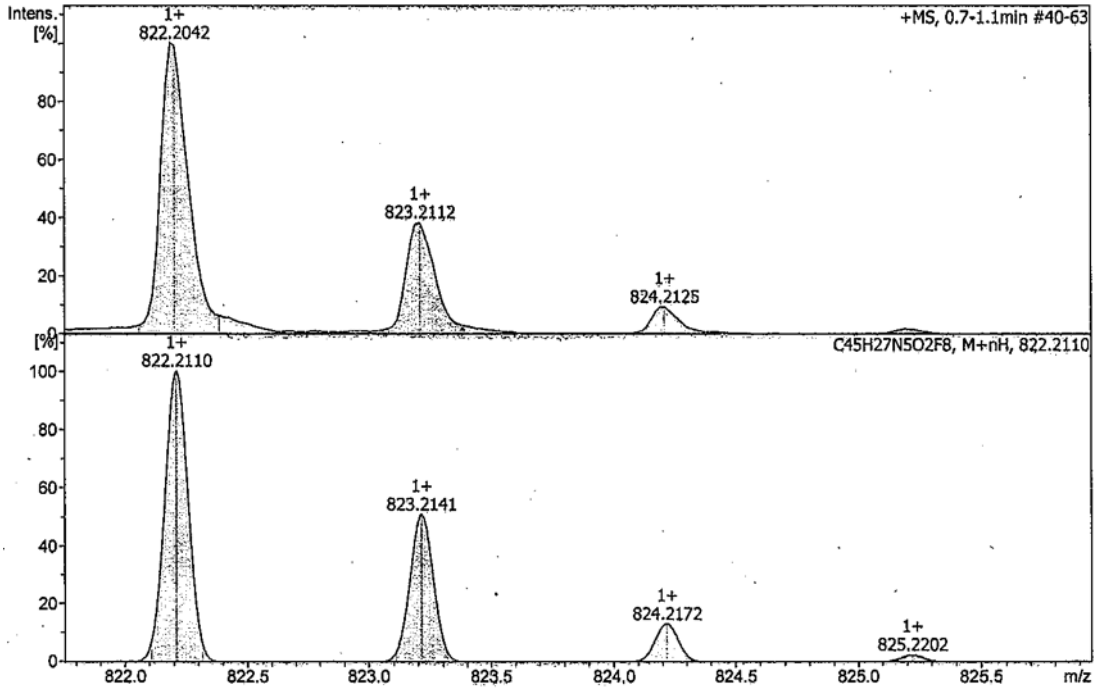

Figure S2-5. Observed (top) and simulated (bottom) HR-APCI-TOF-MS of **6H** *m/z* = 822.2042 (calculated for $[C_{45}H_{28}N_5O_2F_8]^+$; $[M+H]^+$, *m/z* = 821.2110).

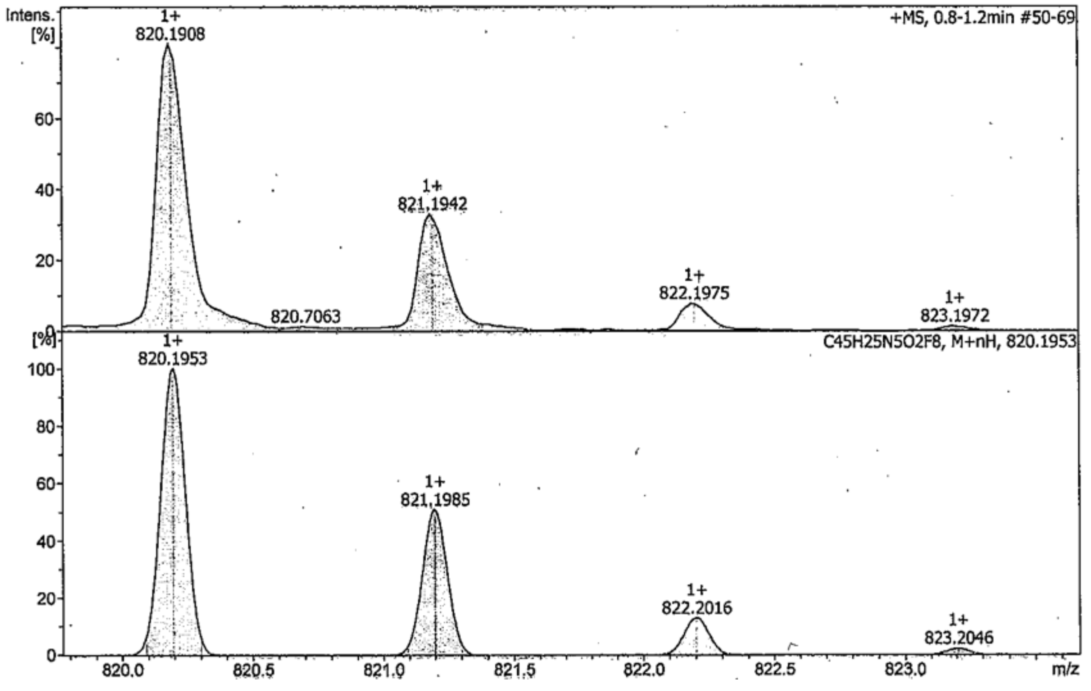

Figure S2-6. Observed (top) and simulated (bottom) HR-APCI-TOF-MS of **7H** *m/z* = 820.1908 (calculated for $[C_{45}H_{25}N_5O_2F_8]^+$; $[M+H]^+$, *m/z* = 820.1953).

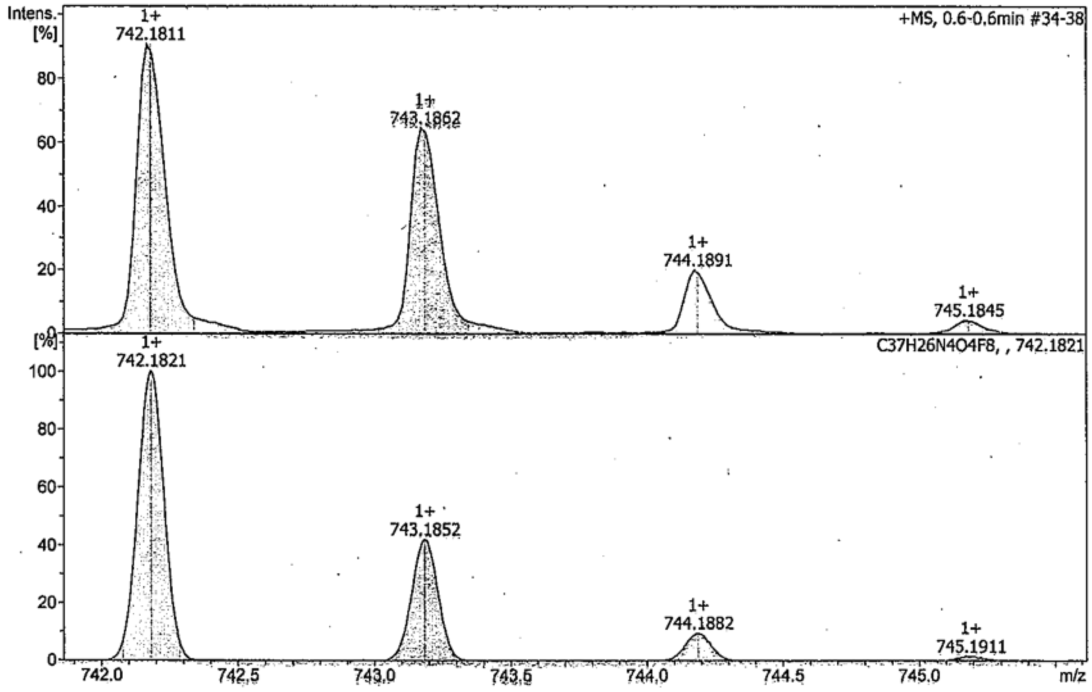

Figure S2-7. Observed (top) and simulated (bottom) HR-APCI-TOF-MS of **8** *m*/*z* = 742.1811 (calculated for $[C_{37}H_{26}N_4O_4F_8]^+$; $[M]^+$, *m*/*z* = 742.1821).

3. UV/Vis Absorption and Fluorescence Spectra

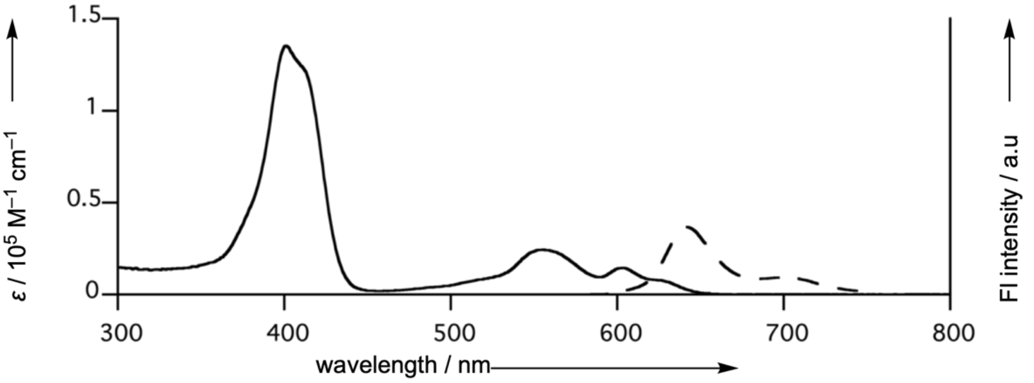

Figure S3-1. UV/Vis absorption (solid) and fluorescence (dashed) spectra of **4**.
UV/Vis (CH_2Cl_2) λ_{max} / nm (ε / 10^5 M^{-1} cm^{-1}) = 401(1.35), 556(0.24), 604(0.14).
FL (CH_2Cl_2) λ_{max} / nm = 642, 700. Φ_F = 9.2%.

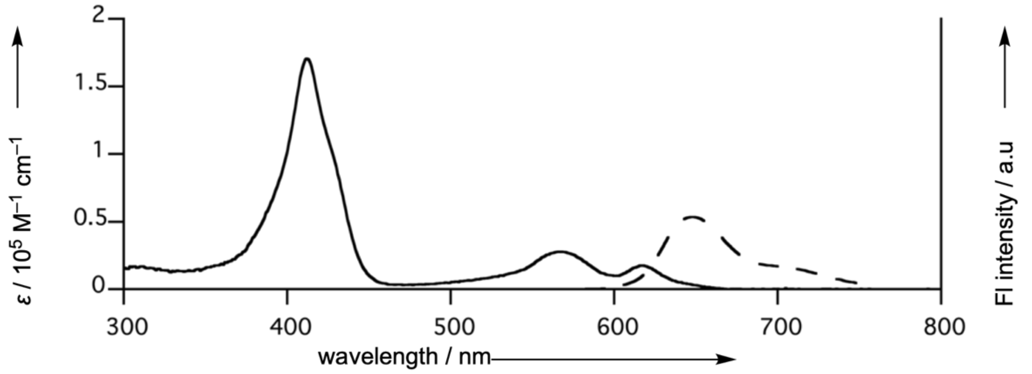

Figure S3-2. UV/Vis absorption (solid) and fluorescence (dashed) spectra of **5H**.
UV/Vis (CH_2Cl_2) λ_{max} / nm (ε / 10^5 M^{-1} cm^{-1}) = 412(1.71), 566(0.28), 616(0.17).
FL (CH_2Cl_2) λ_{max} / nm = 648, 704. Φ_F = 2.3%.

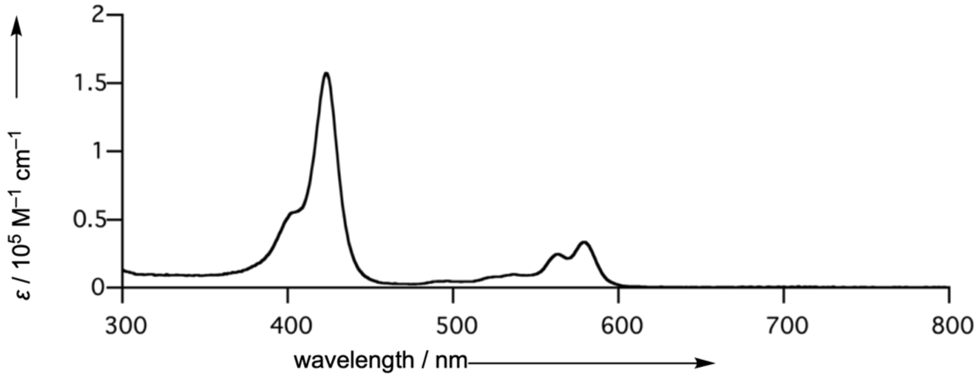

Figure S3-3. UV/Vis absorption spectrum of **5Ag**.
UV/Vis (CH_2Cl_2) λ_{max} / nm (ε / 10^5 M^{-1} cm^{-1}) = 423(1.57), 563(0.25), 579(0.34).

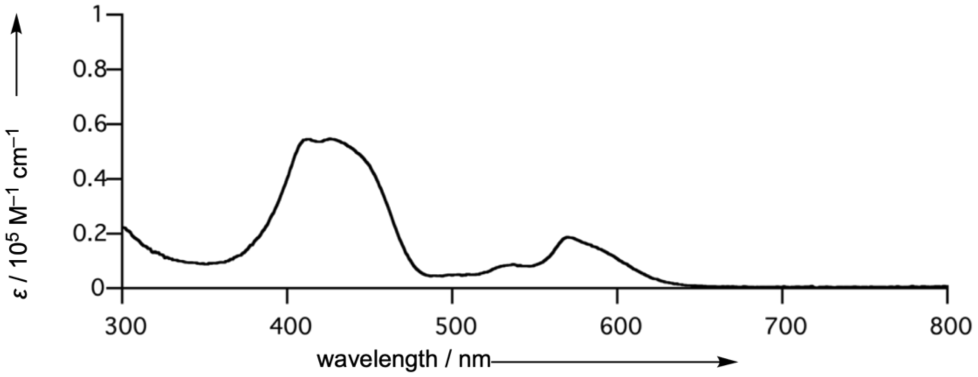

Figure S3-4. UV/Vis absorption spectrum of **6Ag**.
UV/Vis (CH_2Cl_2) λ_{max} / nm (ε / 10^5 M^{-1} cm^{-1}) = 412(0.55), 426(0.55), 570(0.19).

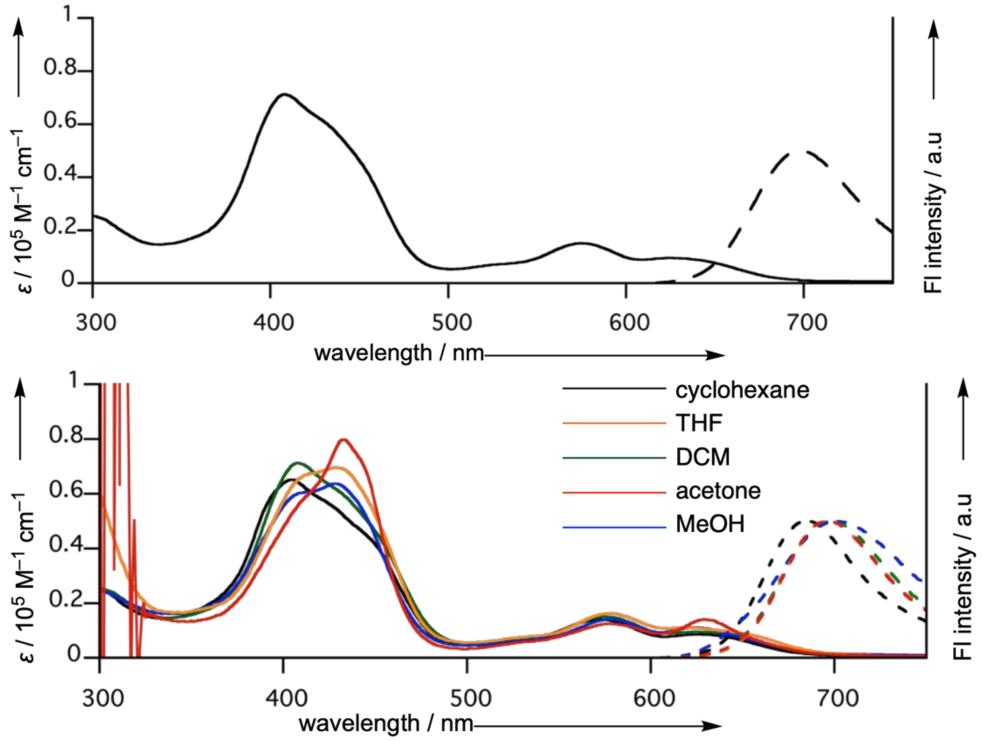

Figure S3-5. UV/Vis absorption (solid) and fluorescence (dashed) spectra of **6H** (top) in CH_2Cl_2 (bottom) in several solvents.

UV/Vis (cyclohexane) λ_{max} / nm (ε / 10^5 M^{-1} cm^{-1}) = 405 (0.65), 573 (0.14), 627 (0.09);
(THF) λ_{max} / nm (ε / 10^5 M^{-1} cm^{-1}) = 429 (0.67), 579 (0.16), 617 (0.11);
(CH_2Cl_2) λ_{max} / nm (ε / 10^5 M^{-1} cm^{-1}) = 408 (0.71), 574 (0.15), 624 (0.10);
(acetone) λ_{max} / nm (ε / 10^5 M^{-1} cm^{-1}) = 433 (0.80), 577 (0.13), 629 (0.14);
(MeOH) λ_{max} / nm (ε / 10^5 M^{-1} cm^{-1}) = 428 (0.64), 578 (0.14), 623 (0.11).

FL (cyclohexane) λ_{max} / nm = 685, Φ_F = 10.3%, τ = 4.63 ns;
(THF) λ_{max} / nm = 696, Φ_F = 11.4%, τ = 3.79 ns;
(CH_2Cl_2) λ_{max} / nm = 699, Φ_F = 10.4%, τ = 3.79 ns;
(acetone) λ_{max} / nm = 696, Φ_F = 10.4%, τ = 3.75 ns;
(MeOH) λ_{max} / nm = 701, Φ_F = 6.9%, τ = 2.67 ns.

The full fluorescence spectra could not be measured because of the instrument limit.

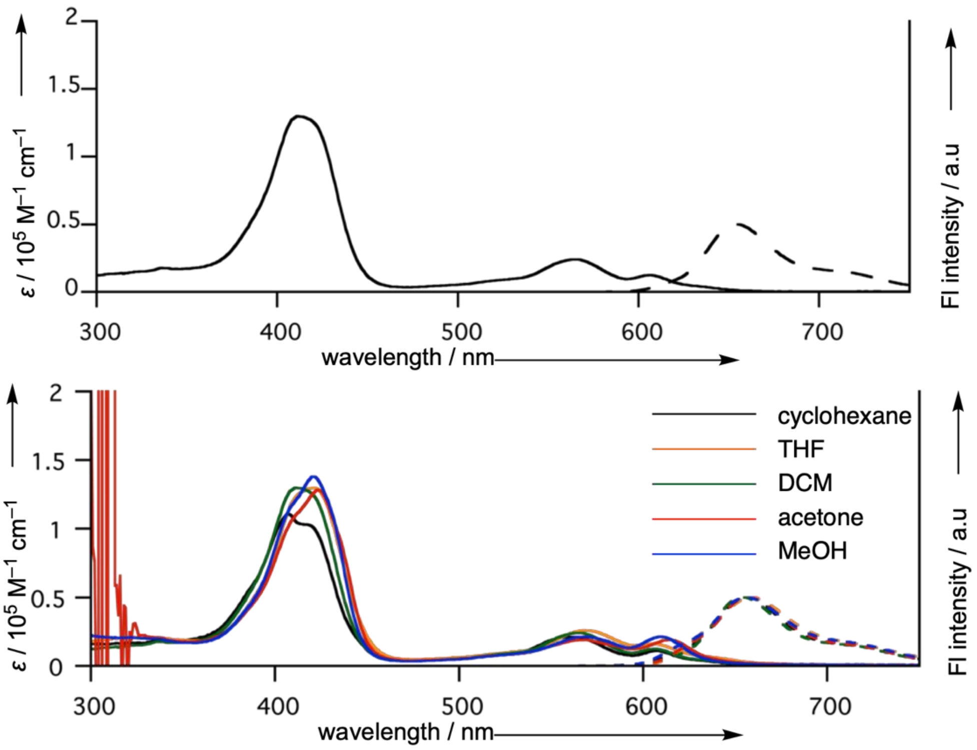

Figure S3-6. UV/Vis absorption spectra (solid) and fluorescence (dashed) spectra of **7H** (top) in CH_2Cl_2 (bottom) in several solvents.

UV/Vis (cyclohexane) λ_{max} / nm (ε / 10^5 M^{-1} cm^{-1}) = 407 (1.11), 417 (1.03), 563 (0.21), 607 (0.11);

(THF) λ_{max} / nm (ε / 10^5 M^{-1} cm^{-1}) = 421(1.30), 568(0.26), 605(0.16);

(CH_2Cl_2) λ_{max} / nm (ε / 10^5 M^{-1} cm^{-1}) = 411 (1.30), 565 (0.24), 606 (0.12);

(acetone) λ_{max} / nm (ε / 10^5 M^{-1} cm^{-1}) = 423 (1.28), 565(0.19), 614(0.19);

(MeOH) λ_{max} / nm (ε / 10^5 M^{-1} cm^{-1}) = 421(1.38), 568(0.21), 609(0.21).

FL (cyclohexane) λ_{max} / nm = 656, Φ_F = 7.5%, τ = 4.28 ns;

(THF) λ_{max} / nm = 659, Φ_F = 10.4%, τ = 3.53 ns;

(CH_2Cl_2) λ_{max} / nm = 654, Φ_F = 9.2%, τ = 3.74 ns;

(acetone) λ_{max} / nm = 657, Φ_F = 9.6%, τ = 3.67 ns;

(MeOH) λ_{max} / nm = 656, Φ_F = 7.6%, τ = 3.69 ns.

Table S1. Absorption and emission details of **6H** and **7H** in various solvents.

Compound	*Solvent*	*Absorption peaks / nm*	*Fluorescence peaks / nm*	*Stokes Shift / cm^{-1}*	Φ_F
**6H**	Cyclohexane	405, 573, 627	685	1050	10.3%
	THF	429, 579, 617	696	1839	11.4%
	CH_2Cl_2	408, 574, 624	699	1720	10.4%
	Acetone	433, 577, 629	696	1530	10.4%
	Methanol	428, 578, 623	701	2236	6.9%
**7H**	Cyclohexane	407, 417, 563, 607	656	1230	7.5%
	THF	421, 568, 605	659	1354	10.4%
	CH_2Cl_2	411, 565, 606	654	1211	9.2%
	Acetone	423, 565, 614	657	1066	9.6%
	Methanol	421, 568, 609	656	1176	7.6%

Table S2. The photophysical parameters of **6H** and **7H** in various solvents.

Compound	*Solvent*	Φ_F	τ / *ns*	k_r / s^{-1}	k_{nr} / s^{-1}
**6H**	Cyclohexane	10.3%	4.63	2.23×10^{7}	1.94×10^{8}
	THF	11.4%	3.79	3.01×10^{7}	2.34×10^{8}
	CH_2Cl_2	10.4%	3.79	2.74×10^{7}	2.36×10^{8}
	Acetone	10.4%	3.75	2.77×10^{7}	2.39×10^{8}
	Methanol	6.9%	2.67	2.58×10^{7}	3.49×10^{8}
**7H**	Cyclohexane	7.5%	4.28	1.75×10^{7}	2.16×10^{8}
	THF	10.4%	3.53	2.95×10^{7}	2.54×10^{8}
	CH_2Cl_2	9.2%	3.74	2.46×10^{7}	2.43×10^{8}
	Acetone	9.6%	3.67	2.62×10^{7}	2.46×10^{8}
	Methanol	7.6%	3.69	2.07×10^{7}	2.51×10^{8}

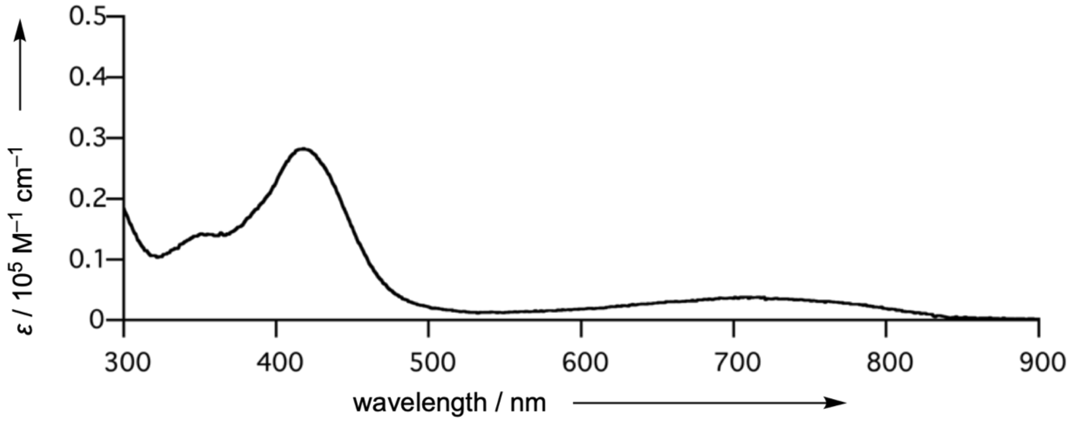

Figure S3-7. UV/Vis absorption spectrum of **8**.

UV/Vis (CH_2Cl_2) λ_{max} / nm (ε / 10^5 M^{-1} cm^{-1}) = 418(0.28), 720(0.02).

4. X-ray Crystallographic Details

Table S3. Crystallographic details of **6Ag**, **6H**, and **8**.

Compound	**6Ag**	**6H**	**8**
Empirical Formula	$C_{45}H_{24}AgF_8N_5O_2$, $2(CH_2Cl_2)$	$C_{45}H_{27}F_8N_5O_2$, CH_2Cl_2	$C_{37}H_{26}F_8N_4O_4$
Fw	1096.41	906.64	742.62
Crystal System	Monoclinic	Monoclinic	Monoclinic
Space Group	$P\ 2_1/c$	$P\ 2_1/n$	$C\ 2/c$
a	19.146(2) Å	12.699(2) Å	30.473(4) Å
b	16.861(2) Å	12.539(4) Å	7.7705(10) Å
c	14.2419(17) Å	24.778(5) Å	29.951(4) Å
α	90°	90°	90°
β	111.296(13)°	94.346(5)°	114.468(3)°
γ	90°	90°	90°
Volume	4283.6(9) $Å^3$	3934.1(16) $Å^3$	6455.2(15) $Å^3$
Z	4	4	8
Density (calculated)	1.700 $g{\cdot}cm^{-3}$	1.531 $g{\cdot}cm^{-3}$	1.528 $g{\cdot}cm^{-3}$
Completeness	0.977	0.991	0.989
Goodness-of-fit (all data)	1.092	1.034	1.100
$R_1(I>2\sigma\ (I))$	0.0425	0.0430	0.0605
wR_2 (all data)	0.1241	0.1084	0.2004
CCDC No.	1893158	1893160	1893159

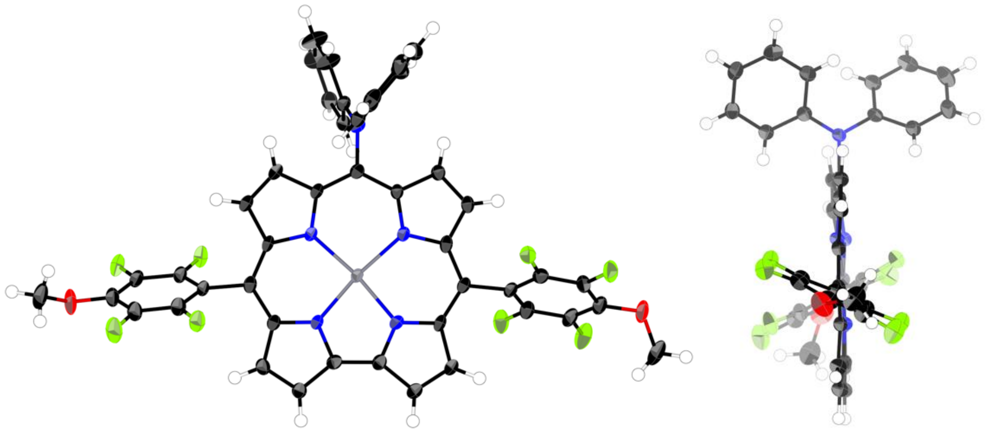

Figure S4-1. X-Ray structure of **6Ag**. (left) Top view and (right) side view. Thermal ellipsoids are shown at the 50% probability level. Solvent molecules are omitted for clarity.

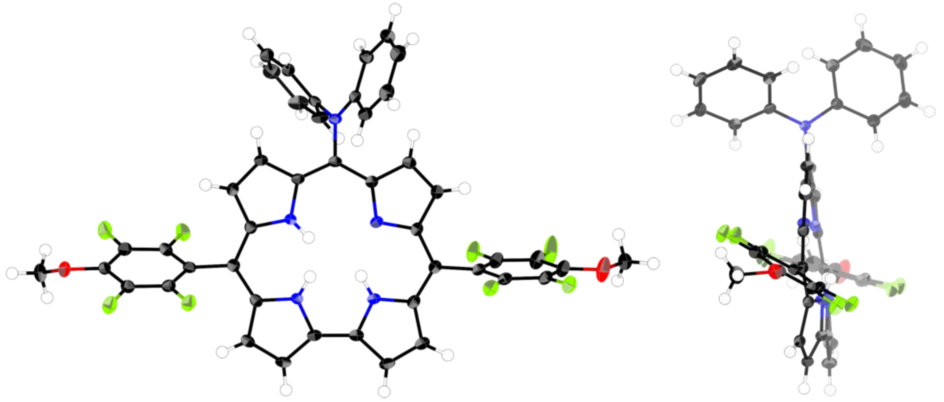

Figure S4-2. X-Ray structure of **6H**. (left) Top view and (right) side view. Thermal ellipsoids are shown at the 50% probability level. Solvent molecules are omitted for clarity.

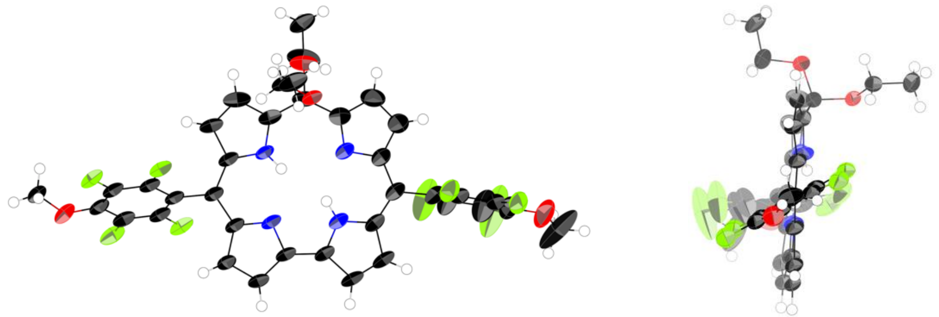

Figure S4-3. X-Ray structure of **8**. (left) Top view and (right) side view. Thermal ellipsoids are shown at the 50% probability level.

5. Cyclic Voltammograms

Conditions: Solvent: 1M nBu$_4$NPF$_6$ solution in dichloromethane. Working electrode: Glassy carbon. Counter electrode: Pt wire. Reference electrode: Ag/AgClO$_4$. Scan rate: 0.05 V/s.

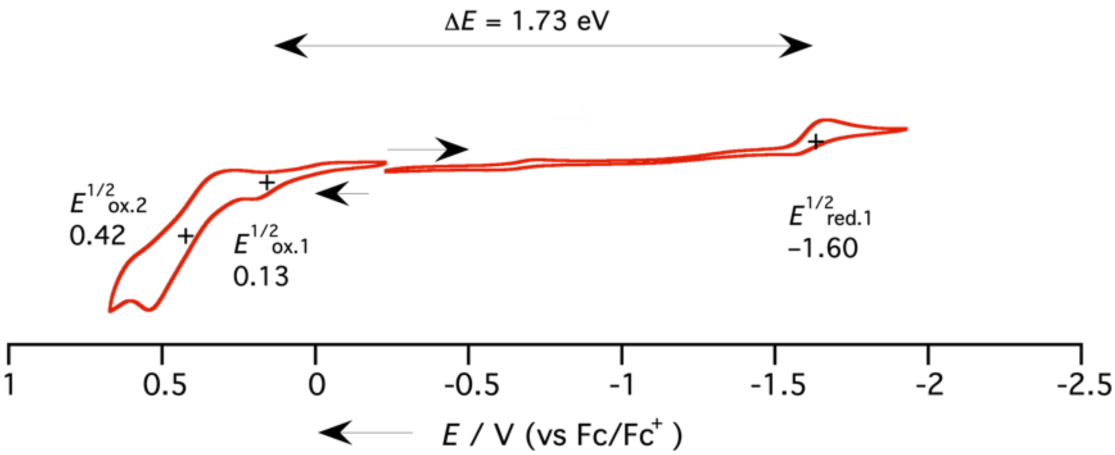

Figure S5-1. Cyclic voltammogram of **4**.

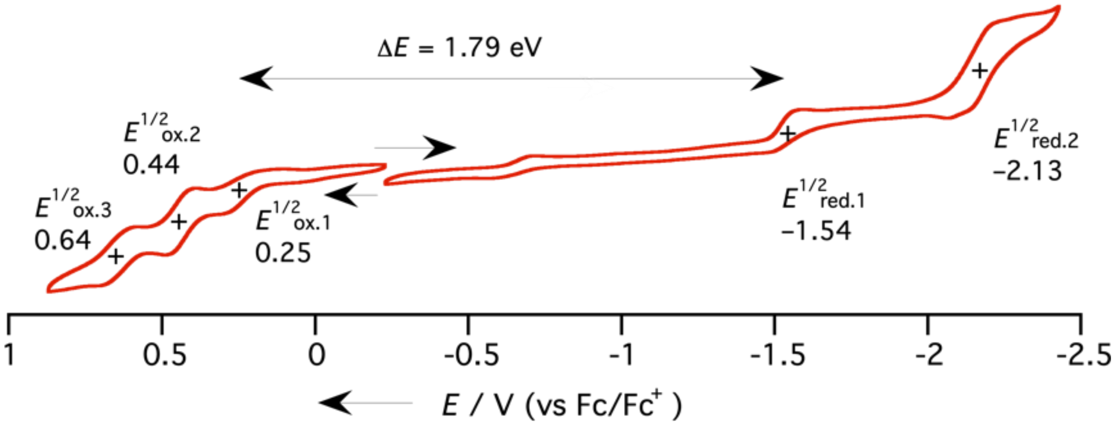

Figure S5-2. Cyclic voltammogram of **5H**.

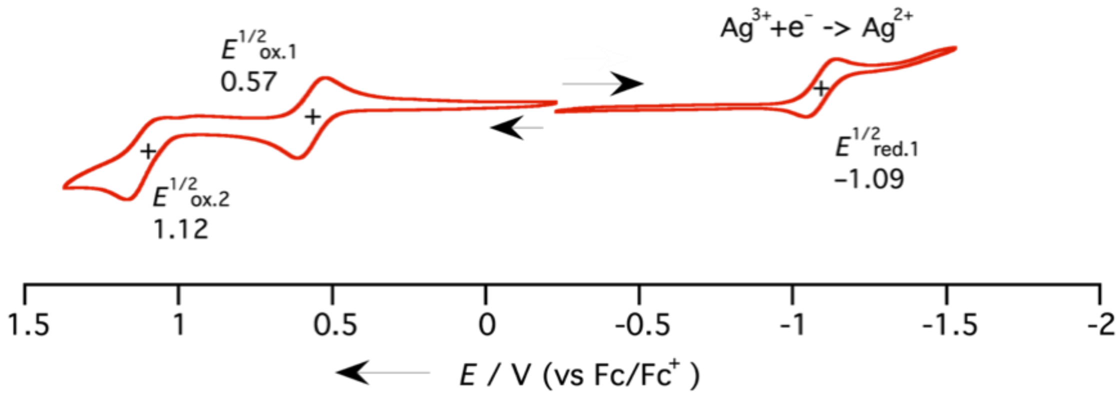

Figure S5-3. Cyclic voltammogram of **5Ag**.

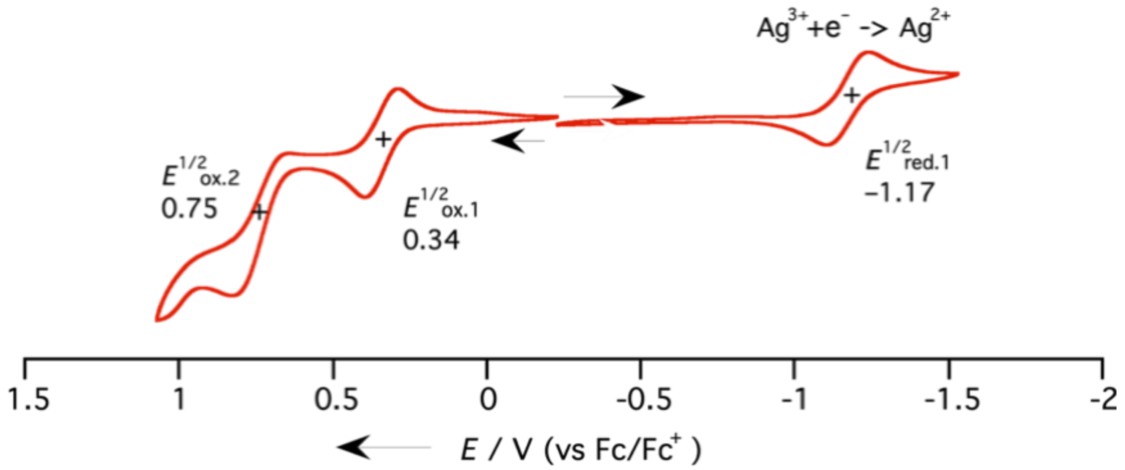

Figure S5-4. Cyclic voltammogram of **6Ag**.

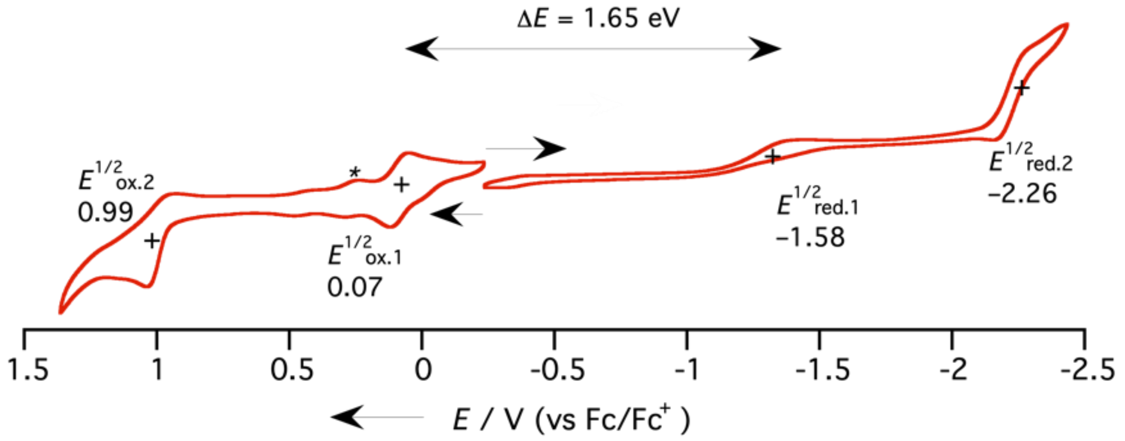

Figure S5-5. Cyclic voltammogram of **6H**.

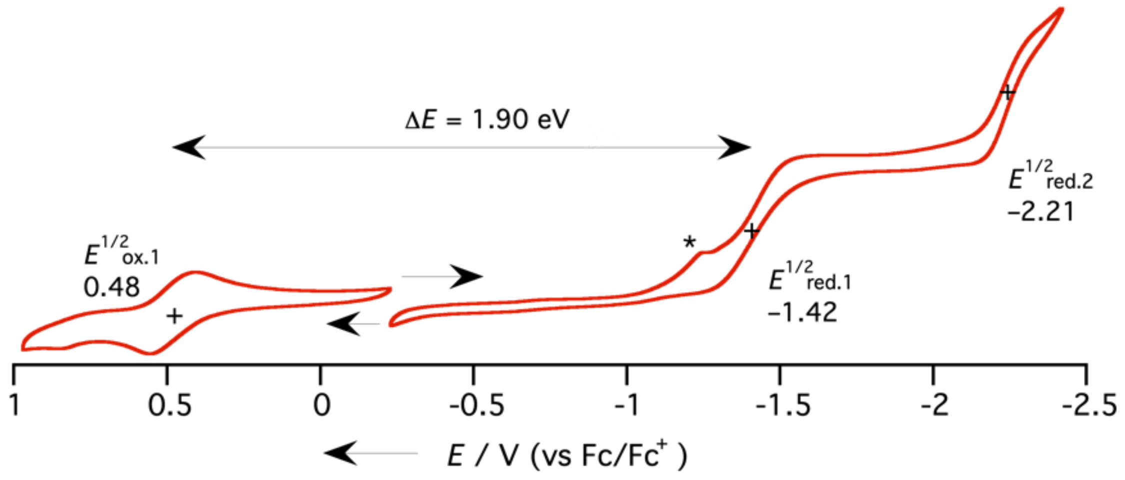

Figure S5-6. Cyclic voltammogram of **7H**.

6. DFT Calculations

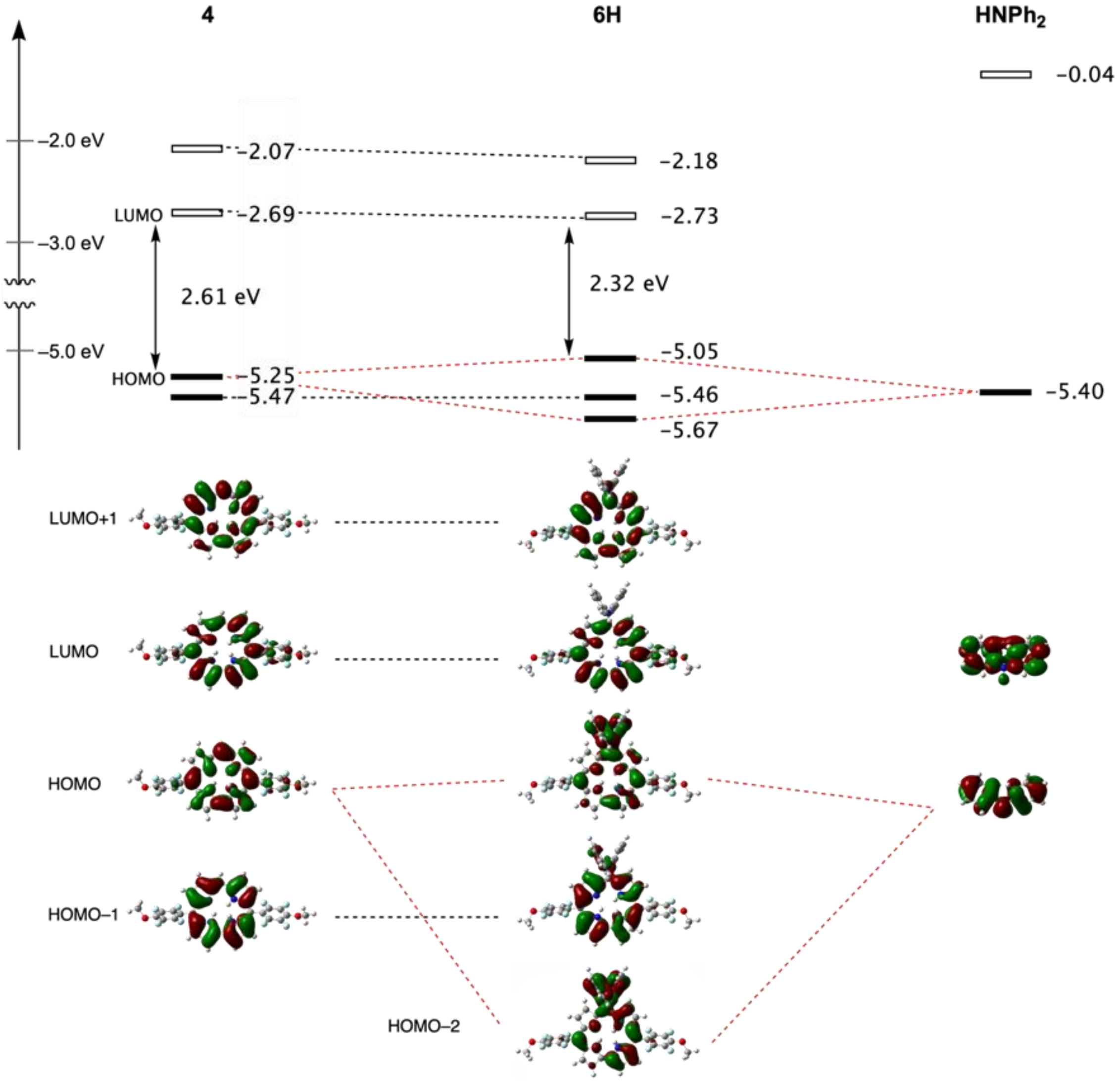

Figure S6-1. MO energy diagrams and Kohn–Sham orbital representations for **4, 6H,** and diphenylamine segment calculated at the B3LYP/6-311G(d,p) level.

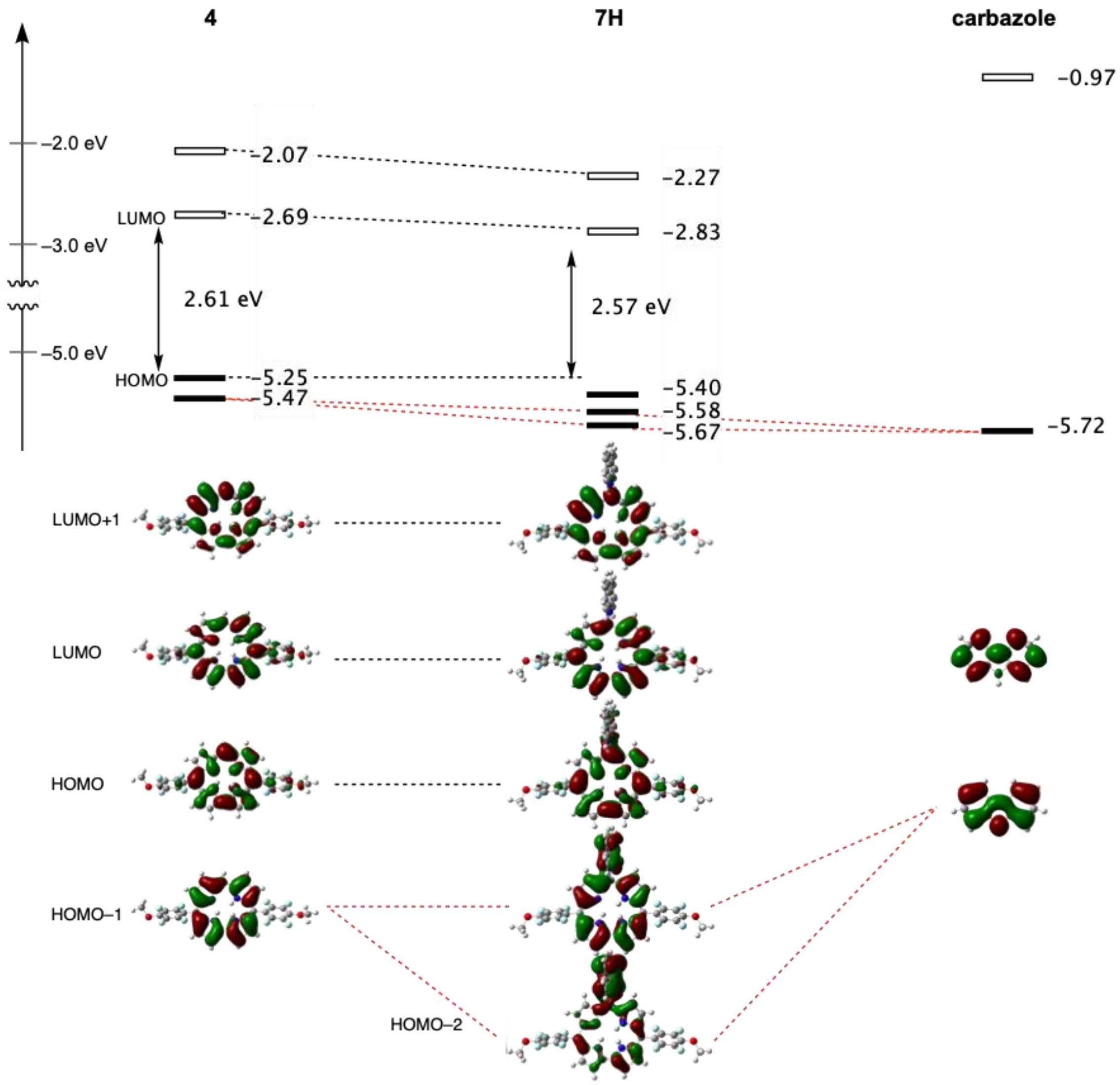

Figure S6-2. MO energy diagrams and Kohn–Sham orbital representations for **4, 7H,** and carbazole segment calculated at the B3LYP/6-311G(d,p) level.

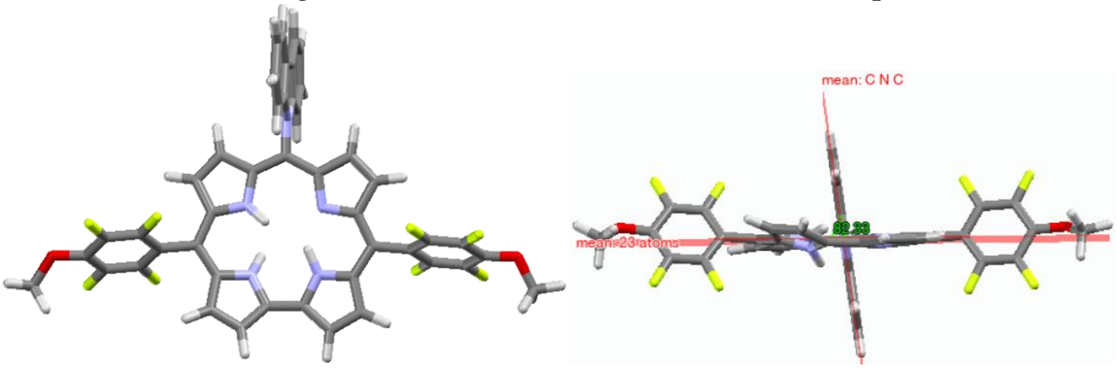

Figure S6-3. Optimized structure of **7H** calculated at the B3LYP/6-311G(d,p) level.

7. Plausible Reaction Mechanism for the Formation of 8

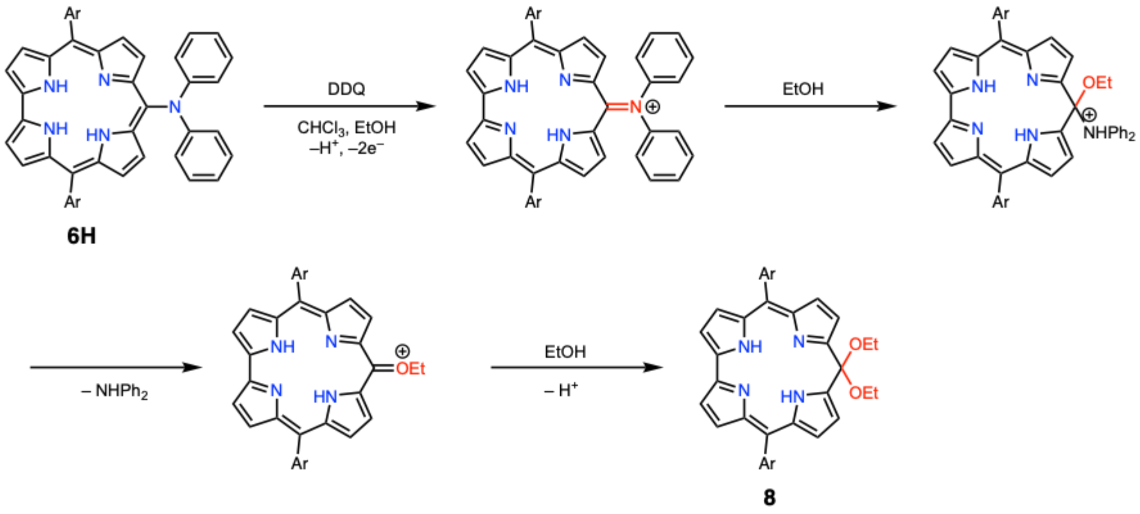

Scheme S7-1. Plausible reaction mechanism for the formation of **8**.

8. References

[S1] Sheldrick, G. M. SHELXT – Integrated space-group and crystal-structure determination. *Acta Cryst*. **2015**, *A71*, 3–8.

[S2] Sheldrick, G. M.; Schneider, T. R. SHELXL: High-resolution refinement. *Methods Enzymol*. **1997**, *277*, 319–343

[S3] Sheldrick, G. M. Crystal structure refinement with SHELXL. *Acta Cryst*. **2015**, *C71*, 3–8.

[S4] Gaussian 16, Revision B.01, Frisch, M. J.; Trucks, G. W.; Schlegel, H. B.; Scuseria, G. E.; Robb, M. A.; Cheeseman, J. R.; Scalmani, G.; Barone, V.; Petersson, G. A.; Nakatsuji, H.; Li, X.; Caricato, M.; Marenich, A. V.; Bloino, J.; Janesko, B. G.; Gomperts, R.; Mennucci, B.; Hratchian, H. P.; Ortiz, J. V.; Izmaylov, A. F.; Sonnenberg, J. L.; Williams-Young, D.; Ding, F.; Lipparini, F.; Egidi, F.; Goings, J.; Peng, B.; Petrone, A.; Henderson, T.; Ranasinghe, D.; Zakrzewski, V. G.; Gao, J.; Rega, N.; Zheng, G.; Liang, W.; Hada, M.; Ehara, M.; Toyota, K.; Fukuda, R.; Hasegawa, J.; Ishida, M.; Nakajima, T.; Honda, Y.; Kitao, O.; Nakai, H.; Vreven, T.; Throssell, K.; Montgomery, J. A., Jr.; Peralta, J. E.; Ogliaro, F.; Bearpark, M. J.; Heyd, J. J.; Brothers, E. N.; Kudin, K. N.; Staroverov, V. N.; Keith, T. A.; Kobayashi, R.; Normand, J.; Raghavachari, K.; Rendell, A. P.; Burant, J. C.; Iyengar, S. S.; Tomasi, J.; Cossi, M.; Millam, J. M.; Klene, M.; Adamo, C.; Cammi, R.; Ochterski, J. W.; Martin, R. L.; Morokuma, K.; Farkas, O.; Foresman, J. B.; Fox, D. J. Gaussian, Inc., Wallingford CT, 2016.
